# HilE represses the activity of the *Salmonella* virulence regulator HilD *via* a mechanism distinct from that of intestinal long-chain fatty acids

**DOI:** 10.1016/j.jbc.2023.105387

**Published:** 2023-10-27

**Authors:** Joe D. Joiner, Wieland Steinchen, Nick Mozer, Thales Kronenberger, Gert Bange, Antti Poso, Samuel Wagner, Marcus D. Hartmann

**Affiliations:** 1 Department of Protein Evolution, Max Planck Institute for Biology Tübingen, Tübingen, Germany; 2 Center for Synthetic Microbiology, Philipps University of Marburg, Marburg, Germany; 3 Department of Chemistry, Philipps University of Marburg, Marburg, Germany; 4 Department of Internal Medicine VIII, University Hospital Tübingen, Tübingen, Germany; 5 Institute of Pharmacy, Pharmaceutical/Medicinal Chemistry and Tübingen Center for Academic Drug Discovery & Development (TüCAD2), Eberhard Karls University Tübingen, Tübingen, Germany; 6 School of Pharmacy, Faculty of Health Sciences, University of Eastern Finland, Kuopio, Finland; 7 Excellence Cluster "Controlling Microbes to Fight Infections" (CMFI), Tübingen, Germany; 8 Interfaculty Institute of Microbiology and Infection Medicine (IMIT), University of Tübingen, Tübingen, Germany; 9 Partner-site Tübingen, German Center for Infection Research (DZIF), Tübingen, Germany; 10 Interfaculty Institute of Biochemistry, University of Tübingen, Tübingen, Germany

**Keywords:** *Salmonella*, pathogenesis, SPI1, T3SS, virulence regulation, HilD, HilE, fatty acids

## Abstract

The expression of virulence factors essential for the invasion of host cells by *Salmonella enterica* is tightly controlled by a network of transcription regulators. The AraC/XylS transcription factor HilD is the main integration point of environmental signals into this regulatory network, with many factors affecting HilD activity. Long-chain fatty acids, which are highly abundant throughout the host intestine, directly bind to and repress HilD, acting as environmental cues to coordinate virulence gene expression. The regulatory protein HilE also negatively regulates HilD activity, through a protein-protein interaction. Both of these regulators inhibit HilD dimerization, preventing HilD from binding to target DNA. We investigated the structural basis of these mechanisms of HilD repression. Long-chain fatty acids bind to a conserved pocket in HilD, in a comparable manner to that reported for other AraC/XylS regulators, whereas HilE forms a stable heterodimer with HilD by binding to the HilD dimerization interface. Our results highlight two distinct, mutually exclusive mechanisms by which HilD activity is repressed, which could be exploited for the development of new antivirulence leads.

*Salmonella enterica* is an enteric pathogen and one of the leading causes of gastrointestinal disease. *Salmonella* spp. adhere to and invade epithelial cells *via* a complex mechanism requiring many virulence factors, most of which are located on five highly conserved horizontally acquired *Salmonella* pathogenicity islands (SPIs) (1, 2, 3). SPI-1 encodes the genes required for the initial invasion of host cells, including numerous effector proteins and a type III secretion system (T3SS-1) injectosome that enables the direct injection of proteins from the bacterial cytoplasm into the host cell (4, 5). These effectors serve several purposes, including inducing changes in the host cell actin cytoskeleton that results in the engulfment of *Salmonella* cells by endocytosis.

To coordinate the sequential expression of different virulence genes according to the stage of the infection process, expression of SPI genes is tightly regulated. The transcriptional regulator HilA activates the expression of the *prg*/*org* and *inv*/*spa* operons, which encode the structural components of the T3SS-1, and the genes encoding several of the effector proteins secreted through T3SS-1 (6, 7, 8). Expression of *hilA* is in turn controlled by the action of three AraC/XylS transcription regulators: HilD, HilC, and RtsA, which bind to overlapping sites within the *hilA* promoter to activate expression (9, 10). The AraC/XylS family is defined by a highly conserved DNA-binding domain (DBD) containing two helix-turn-helix (HTH) motifs, which form direct contacts with DNA (11, 12, 13). HilD, HilC, and RtsA all have a two-domain structure comprising an N-terminal regulatory domain and a C-terminal DBD, which is the most common domain organization of AraC/XylS family proteins (13); examples include the regulators ToxT and Rns, for which full-length structures have been experimentally determined (14, 15). Each of HilD/HilC/RtsA is able to activate not only *hilA*, but also its own promoter and that of the other two regulators, forming a complex feed-forward loop to activate SPI-1 expression (Fig. 1) (16). HilD is the most prominent activator of *hilA*, with HilC and RtsA serving to amplify *hilA* transcription (16, 17).

**Figure 1:**
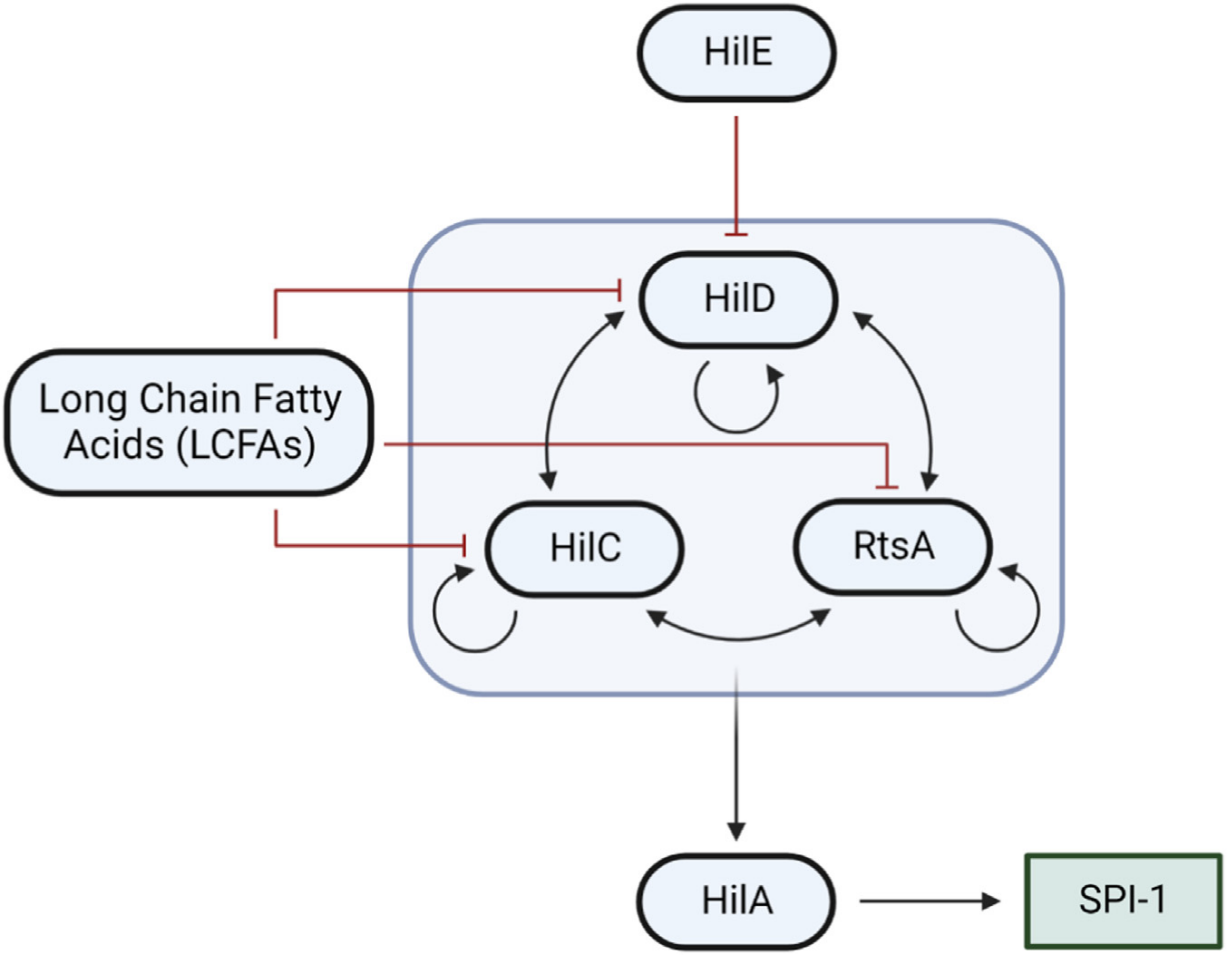
**Simplified model of the SPI-1 regulatory network.***Black arrows* indicate activation and *red lines* with blunt ends represent repression.

HilD is additionally the main integration point of environmental signals into the SPI-1 regulatory network, as many regulatory factors affect *hilD* transcription, translation, or HilD activity to regulate *hilA* activation (18). HilD/HilC/RtsA can accommodate a range of small molecules, which regulate their ability to bind to target DNA. These include long-chain fatty acids (LCFAs) and bile acids present in the gut, which *Salmonella* utilize to sense their intestinal location and coordinate the expression of virulence genes at specific locations where invasion can occur (19, 20, 21, 22). Other compounds have also been shown to bind to these regulators to inhibit *hilA* expression and *Salmonella* invasion, highlighting the potential of these regulators as targets of novel antipathogenic compounds (23, 24).

One of the most important negative regulators of HilD is the regulatory protein HilE. HilE is a homolog of hemolysin-coregulated protein (Hcp), a key structural component of the type VI secretion system, and specifically represses HilD activity through a protein-protein interaction (25, 26, 27). However, the mechanism and structural basis of this interaction remain elusive. While one study reported that HilE negatively affects HilD dimerization to inhibit the DNA-binding of HilD (26), another study suggested that HilD and HilE form a large protein complex without compromising the integrity of the HilD homodimer (27).

Here, we used a range of biochemical and biophysical methods to elucidate and compare the regulation of the HilD protein by LCFAs and HilE. We show that LCFAs bind to, and regulate, HilD through a mechanism comparable to other AraC/XylS regulators of virulence genes. HilE forms a stable heterodimer with HilD, disrupting HilD homodimerization and preventing HilD from binding to target DNA. Our results highlight the different biochemical mechanisms that exist to repress HilD activity, and a unique mechanism for the regulation of AraC/XylS transcription factors.

## Results

### Structural and dimerization characteristics of HilD

**Figure 2:**
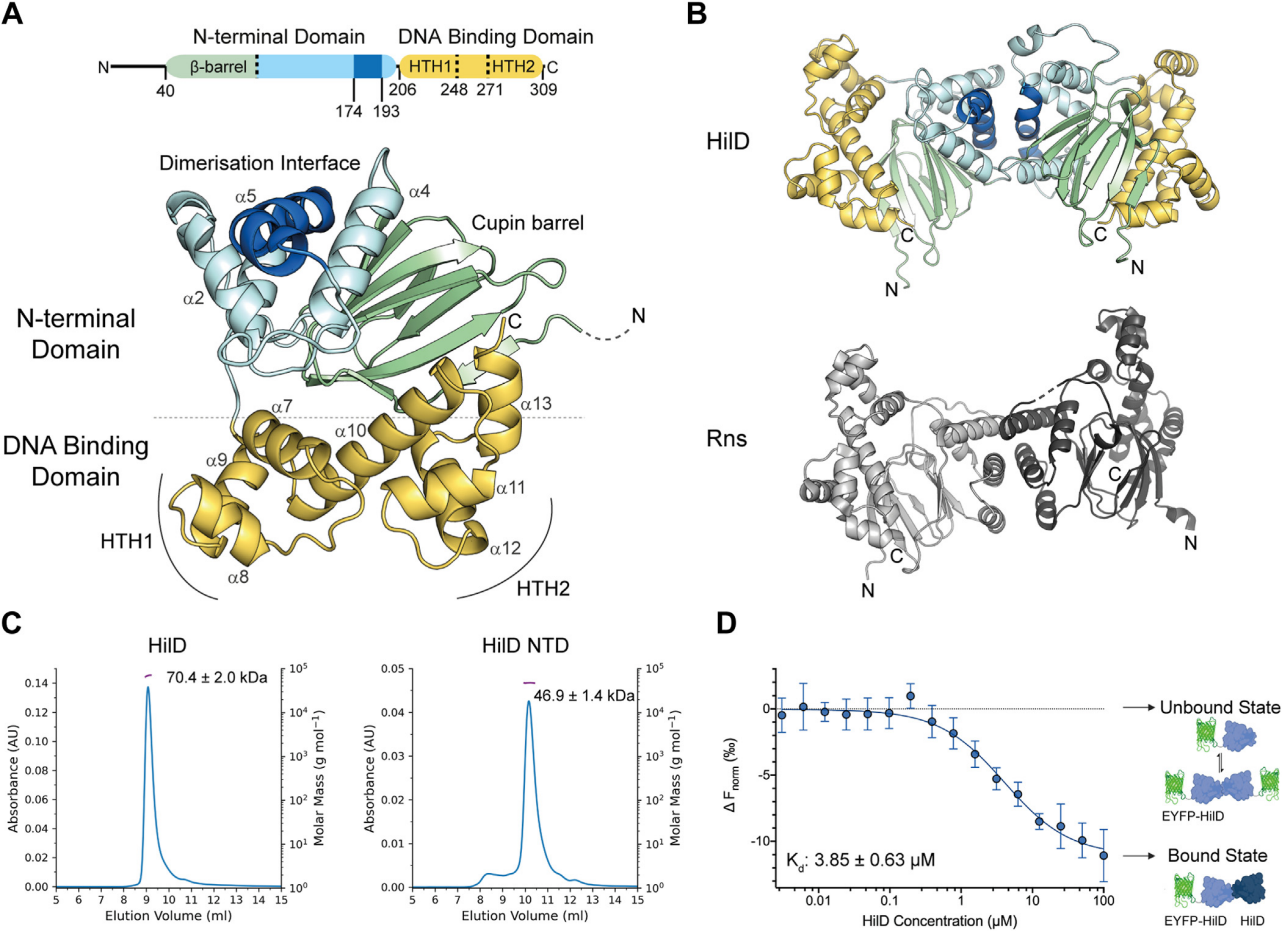
**HilD forms homodimers reminiscent of other AraC/XylS transcription regulators.***A*, AlphaFold2 model of HilD. The N-terminal domain is colored in *cyan*, with the cupin barrel and reported dimerization helix highlighted in *green* and *dark blue*, respectively. The DNA binding domain is colored in *yellow*, with the helices constituting this domain and the two HTH motifs labeled. *B*, AlphaFold2 model of the HilD homodimer (*top*) and the crystal structure of the Rns homodimer (PDB: 6XIV) (*bottom*). HilD residues 1 to 35 are removed in both (*A* and *B*) clarity. *C*, SEC-MALS profiles of full-length and the N-terminal domain (NTD) of HilD. Calculated molecular weight values correspond to three repeat experiments. *D*, HilD dimerization measured by MST. Unlabeled HilD protein (3.05 nM to 100 μM) was incubated with 50 nM EYFP-HilD, which exists as a mixture of monomers and dimers at this concentration (Fig. S2). Data represent mean ± SD of four replicates.

The structural characterization of AraC/XylS proteins is challenging due to their poor solubility at higher concentrations (28), and an experimental structure of HilD remains elusive. The predicted structure of HilD, retrieved from the AlphaFold database (AlphaFold EBI ID P0CL08), concurs with the expected domain organization (Fig. 2*A*) and is comparable to other AraC/XylS proteins for which full-length structures have been experimentally determined (14, 15, 29). The N-terminal domain (NTD) contains a cupin barrel structure, which has been shown to form the fatty acid binding site in the AraC/XylS family members ToxT and Rns, as well as a number of helices that form the reported dimerization interface. The C-terminal DBD is comprised of 7 α-helices (α7-α13), which constitute two HTH motifs connected by an α-helical linker (helix α10). The N-terminus of HilD (residues 1–35) was predicted with very low confidence (pLDDT < 50%) in the AlphaFold model, indicative of disorder, and hence is hidden in all protein figures for clarity.

HilD is known to form both homodimers and heterodimers with the other SPI-1 regulators HilC and RtsA (30), and we modeled the HilD homodimer using AlphaFold multimer (31). The predicted homodimer topology of HilD is comparable to that of the AraC/XylS proteins Rns and ExsA for which the dimeric structure has been experimentally determined (15, 32) (Fig. 2*B*). We used multi-angle light scattering (MALS) coupled to size-exclusion chromatography (SEC) to confirm that our purified full-length HilD exists exclusively as a dimer in solution (Figs. 2*C* and S1). We also purified a construct lacking the DNA binding domain (HilD NTD, residues 7–206) that similar to full-length HilD appears dimeric during SEC-MALS runs (Fig. 2*C*). This is consistent with previous data showing that the NTD is responsible for HilD dimerization, which is primarily mediated by helix α5 (formed by residues 180–192) at the center of the dimerization interface (30).

We conceived a microscale thermophoresis (MST) assay to quantify the homodimerization of HilD, in which HilD was fused to an N-terminal EYFP tag for detection. EYFP-HilD, at a constant concentration of 50 nM, was then incubated with varying concentrations of unfused HilD. A dose-dependent reduction in normalized fluorescence yielded an equilibrium dissociation constant for HilD dimerization, *K*_*d*,dimer_, of 3.85 ± 0.63 μM (Fig. 2*D*). Collectively, these data suggest that HilD exhibits the typical characteristics of AraC/XylS protein family members.

### HilD binds a range of fatty acids

**Figure 3:**
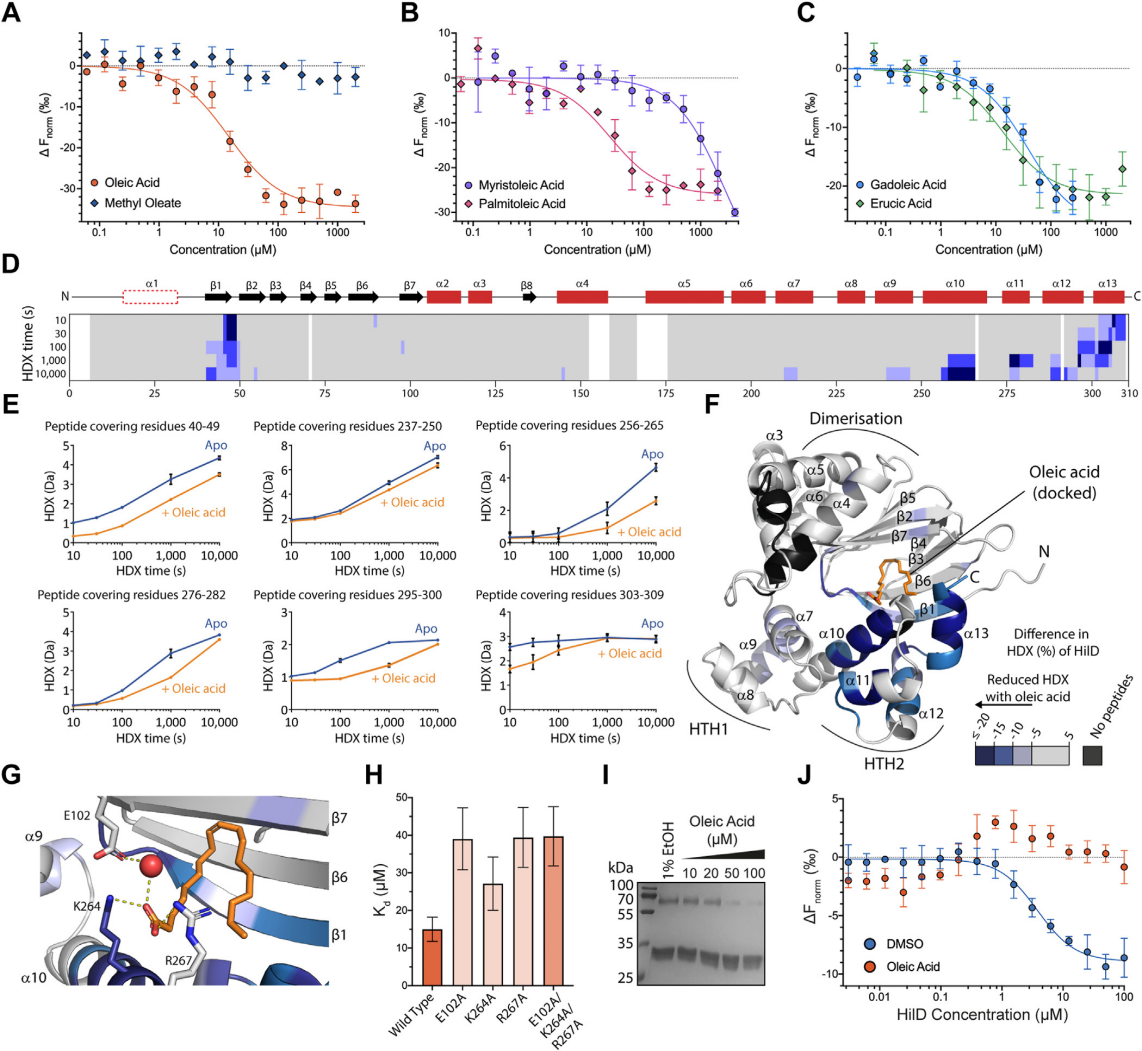
**Long-chain fatty acids bind to a conserved binding pocket in HilD.***A*–*C*, MST dose-response curves for fatty acid binding to HilD. Data represent the mean ± SD of four replicates. Calculated binding affinities are displayed in Table 1. *D*, the difference in HDX between oleic acid-bound and apo HilD projected on its amino acid sequence. Different tones of *blue* reflect reduction in HDX of HilD in presence of oleic acid. The HilD secondary structure is schematically depicted above. *E*, representative HilD peptides displaying changes in HDX with respect to oleic acid. Data represent the mean ± SD of three replicates. *F*, the oleic acid-dependent HDX changes were projected onto the structural model of HilD with the oleic acid binding site inferred by molecular docking of the ligand. *G*, detailed view of the oleic acid binding pocket shown in (*F*). Residues predicted to form specific interactions with oleic acid are displayed as *sticks*, with hydrogen bonds shown as *yellow dashes* and the bound water molecule represented as a *red sphere*. *H*, binding affinities of oleic acid to HilD point mutants, as determined by MST. Bars and error bars represent the mean and SD of four independent replicates. Full binding curves are shown in Fig. S5. *I*, BS^3^ cross-linking of HilD (10 μM) in the presence of increasing concentrations of oleic acid. *J*, MST dose-response plot for the homodimerization of HilD, whereby EYFP-HilD was preincubated with 1% DMSO (*blue*), or 100 μM oleic acid (*orange*). The *K*_*d*,dimer_ was determined from changes in thermophoresis at an MST on-time of 1.5 s. Data represent the mean ± SD of four replicates.

HilD is capable of binding a range of different LCFAs and a number of residues were previously reported to contribute to this interaction (20). Using MST, we first determined the affinity of oleic acid, which has previously been shown to bind to HilD to repress *hilA* expression (19, 20, 33), to HilD and obtained a *K*_*d*_ of 14.98 ± 3.21 μM for this interaction (Fig. 3*A*).

We then determined the binding affinity of a number of other unsaturated fatty acids, to establish which structure-related properties of these ligands are critical for binding to HilD. We first examined the effect of varying the chain length of LCFAs containing a *cis*-9 double bond, as in oleic acid. Palmitoleic acid (C16) and gadoleic acid (C20) bound to HilD with similar affinities (27.62 ± 8.70 and 27.60 ± 10.67 μM, respectively), comparable to that of oleic acid, highlighting the flexibility of fatty acid chain length in binding (Table 1 and Fig. 3, *B* and *C*). Myristoleic acid (C14) displayed only very weak binding (K_d_ > 2 mM), signifying 16 carbon atoms as the minimum chain length of fatty acids that is required for efficient binding to HilD. Erucic acid (C22, *cis*-13 unsaturation), which has an increased chain length between the *cis*-double bond and the carboxylic acid head group relative to oleic acid, also bound to HilD with a similar affinity to oleic acid (12.80 ± 3.33 μM) (Fig. 3*C*).

**Table 1:** Affinity values for the binding of LCFAs to HilD

Lipid	Shorthand nomenclature	K_d_ ± SD (μM)
Myristoleic acid	9Z-14:1	2606 ± 1082
Palmitoleic acid	9Z-16:1	27.62 ± 8.70
Oleic acid	9Z-18:1	14.98 ± 3.21
Gadoleic acid	9Z-20:1	27.60 ± 10.67
Erucic acid	13Z-22:1	12.80 ± 3.33
Methyl oleate	9Z-18:1	-

*K*_*d*_ values were calculated from changes in normalized fluorescence (ΔF_norm_) at an MST on-time of 1.5 s with increasing ligand concentrations. *K*_*d*_ values and standard deviation were calculated using the MO.Affinity Analysis v2.3 software (NanoTemper Technologies GmbH) from four independent replicates.

Although we could not determine the binding affinity of corresponding *trans*-unsaturated or longer chained LCFAs due to the low solubility of these compounds, we observed the binding of nervonic acid (C24, *cis*-15 unsaturation) and elaidic acid (the *trans-*isomer of oleic acid) to HilD in electrophoretic mobility shift assays (EMSAs) (Fig. S3, *A* and *B*). Elaidic acid appears to bind to HilD with similar affinity to oleic acid. Nervonic acid inhibited HilD DNA binding at all tested concentrations, and MST runs performed in the presence of Pluronic F-127 implicated it to have a similar binding affinity to erucic acid (see Supporting information, Table S1 and Fig. S4). These combined results imply that the position of this central double bond is not a requirement for binding, supported by previous findings that *cis*-2-unsaturated fatty acids, which lack a double bond in the center of the hydrocarbon chain, bind to HilD with high affinity (33). HilD is able to accommodate the binding of a range of fatty acids with a chain length of up to at least 24 carbon atoms.

To investigate whether specific interactions involving the carboxylic acid head group are important for LCFA binding, we compared the binding of oleic acid with its corresponding methyl ester (Fig. 3*A*). Methyl oleate did not bind to HilD, in agreement with a previous study showing methyl esters of *cis*-2-unsaturated fatty acids showed reduced potency in repressing *hilA* (33), highlighting the importance of the carboxylic acid head group in ligand binding.

### LCFAs bind to HilD in a comparable manner to other AraC/XylS proteins

To experimentally probe the LCFA binding pocket in HilD, we used hydrogen–deuterium exchange mass spectrometry (HDX-MS). HDX-MS detects changes in the accessibility of backbone amide hydrogens, which undergo exchange with deuterium in deuterated buffers on a time scale measurable by MS, and thus provides snapshots of a protein’s higher order structure and changes therein by for example, ligand binding. HDX-MS was performed on HilD in both the absence and presence of oleic acid. We mapped detected changes in HDX upon incubation with oleic acid onto the predicted structure of HilD to identity the location of the binding pocket (Fig. 3, *D*–*F*). Decreased HDX was observed over the entire DNA binding domain, but most pronounced at the C-terminal portion of helix α10 and helices α11 to α13, and β-strand β1, which altogether line the cavity provided by the β-barrel (Fig. 3*F*). Only minor changes were apparent for the other β-strands forming the barrel structure, which may be reasoned by the intrinsically very low HDX rate of these entities (Dataset S1). Mildly altered HDX in α7 and α9 (HTH1) may be a consequence of the change in α10, preventing the independent rotation of the two HTH motifs with respect to one another. Collectively, the vicinity of the strongest HDX decreases suggests the β-barrel/HTH2 interface as the oleic acid binding pocket, in agreement with the fatty acid binding pocket in ToxT (14) and Rns (15), and computational docking of oleic acid to HilD (Fig. 3*F*). In this model, specific polar interactions are predicted between the carboxyl head group of oleic acid with residues K264 and R267, which are located on α10 of the DBD within a region of decreased HDX, and water-mediated interactions with residue E102 (Fig. 3*G*). To experimentally validate the importance of these residues in LCFA binding, we mutated each of them to alanine and determined the binding affinity of oleic acid to each of these mutants (Figs. 3*H* and S5). Mutants E102A and R267A displayed decreased affinity for oleic acid (39.05 ± 8.26 and 39.44 ± 7.98 μM, respectively). Mutation of residue K264 resulted in a less significant decrease in binding affinity (27.11 ± 7.08 μM), while the triple alanine mutant (39.71 ± 7.90 μM) showed no further decrease in binding compared to the individual E102A and R267A mutants. Taken together, these results suggest that the binding of LCFAs to HilD is driven mostly by hydrophobic interactions; however, polar interactions also play a role in ligand binding specificity.

We also found that the binding of oleic or palmitoleic acid increased the melting temperature of HilD, whereas the non-binding myristoleic acid did not affect the melting temperature of HilD (Fig. S3, *E* and *F*). This is similar to the effect observed for fatty acid binding to Rns (15) and is consistent with our model showing that oleic acid forms specific interactions with residues on both domains, confining HilD to a more rigid structure.

The binding of small molecules has previously been shown to disrupt the dimerization of both HilD (22) and ToxT (34), and we performed bis(sulfosuccinimidyl)suberate (BS^3^) cross-linking of HilD after incubation with different LCFAs, to investigate their effects on HilD homodimerization. Oleic acid decreased the levels of the cross-linked HilD dimer (Fig. 3*I*), in a comparable manner to that previously reported for the bile acid chenodeoxycholic acid (22). Other LCFAs that bind to HilD had similar disruptive effects on HilD homodimerization, while methyl oleate did not (Fig. S3, *C* and *D*). We also used the MST dimerization assay (Fig. 2*D*) to verify the effect of oleic acid on HilD homodimerization. Whilst dimethyl sulfoxide (DMSO) had no significant effect on HilD dimerization (*K*_*d*,dimer_: 4.33 ± 0.72 μM), when EYFP-HilD was incubated with oleic acid (100 μM), the formation of heterodimers between EYFP-HilD and unfused HilD was completely abolished (Fig. 3*J*). Our results indicate that LCFAs disrupt the dimerization of HilD to prevent HilD from binding to target DNA, in a similar manner as reported for ToxT (35, 36).

### HilE does not form higher order oligomers

An experimentally determined structure of HilE remains elusive; however, its predicted structure is comparable to that of Hcp family proteins, comprising a tight β-barrel domain with a single α-helix (residues 58–68) located on one side of the β-barrel (Fig. S6*D*). Although the C-terminus is predicted with low confidence in the AlphaFold model, indicative of disorder, other prediction servers modeled the C-terminus as an additional β-strand, as observed in the structures of other Hcp proteins (Fig. S6*D*).

Other Hcp family proteins assemble into hexameric ring structures. Based on its predicted structural similarity to these proteins, HilE is also postulated to form oligomers and it was previously reported that HilE may inhibit HilD activity through the formation of a large protein complex with HilD (27). We recombinantly expressed and purified HilE to homogeneity in high yields (Fig. S6*A*) and determined its oligomerization state using SEC-MALS. Surprisingly, HilE was found to exist exclusively as monomers in solution (Fig. S6*C*). We expressed several different HilE constructs with different purification tags confirming that the additional residues introduced by tags at either terminus were not inhibiting ring formation (Fig. S7). An extended loop region, which can be defined from the structural alignment of Hcp proteins, has previously been shown to be crucial for the assembly of Hcp proteins into hexameric rings and subsequent ring stacking during nanotube formation (37). Mutants of *Salmonella* Hcp2, containing mutations within this loop region, were defective in ring formation (38). This loop is also notably shorter in HilE (formed by residues T34-Y45) than in other Hcp-like proteins (Fig. S6, *D* and *E*), explaining the observation that HilE does not appear to form hexameric rings.

### HilE forms a stable heterodimer with HilD

**Figure 4:**
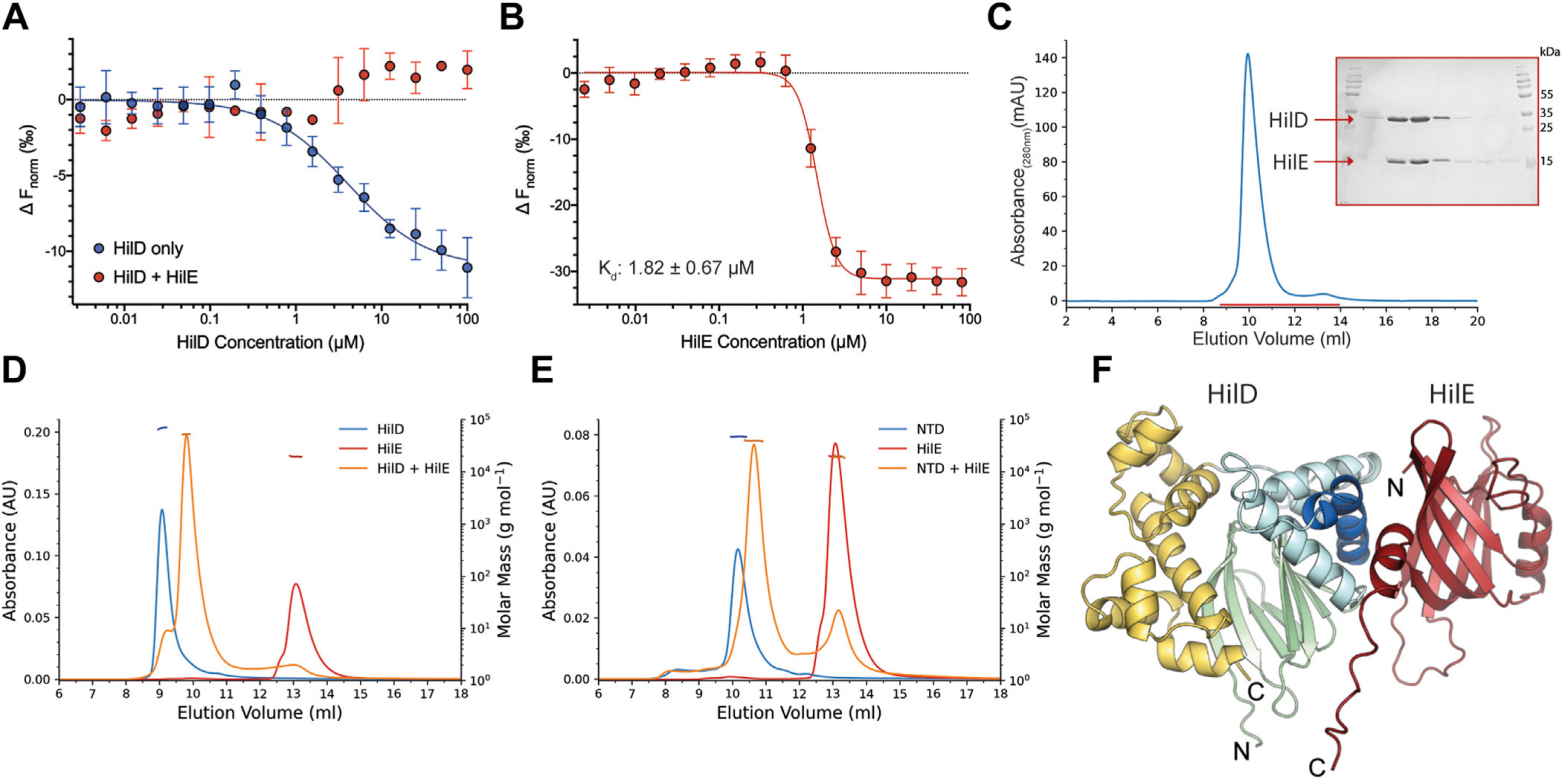
**HilD and HilE form a stable heterodimer.***A*, homodimerization of HilD monitored by MST. Unlabeled HilD protein (3.05 nM to 100 μM) was incubated with 50 nM EYFP-HilD, in the presence (*red*) or absence (*blue*, Fig. 2*D*) of 10 μM HilE. *K*_*d*,dimer_ was determined from changes in thermophoresis at an MST on-time of 1.5 s, and data represents mean ± SD from n = 4 (HilD only) or n = 3 (HilD + HilE) replicates. *B*, MST dose-response curve for the binding of HilD and HilE. HilE was titrated against EYFP-HilD (50 nM) and the *K*_*d*_ calculated from changes in thermophoresis at 1.5 s on-time (5 repeat experiments). *C*, elution profile for the purification of the HilD-HilE complex using a S75 10/300 increase size-exclusion chromatography column. Selected fractions, highlighted by the *red line* in the elution trace, were loaded to an SDS gel and stained with Coomassie. *D* and *E*, SEC-MALS analysis of (*D*) HilD and HilE and (*E*) HilD NTD and HilE. Protein concentrations of 100 μM were used for all runs. Left *x*-axis shows UV absorbance measured at 280 nm; *right x*-axis shows the calculated molecular weight values from light scattering, highlighted by horizontal *dashes*, with values displayed in Table 2. *F*, highest ranked model of the HilD-HilE heterodimer was predicted using AlphaFold Multimer. HilD is colored as in Figure 1, with HilE colored in *red*.

HilE has previously been shown to prevent HilD from binding to target DNA; however, whether HilE also affects HilD dimerization has been subject to debate (26, 27). We found that HilD dimerization was no longer observed in our MST-based dimerization assay when EYFP-HilD was first incubated with excess HilE (Fig. 4*A*), akin to the effect seen for oleic acid. This suggests that HilE negatively regulates HilD activity by inhibiting HilD dimerization and subsequently preventing DNA binding. We determined the affinity of the HilD-HilE interaction by MST, showing that HilE binds to HilD with a *K*_*d*_ of 1.82 ± 0.67 μM (Fig. 4*B*). To further characterize the interaction between HilD and HilE, we next performed a gel-filtration assay to confirm the two proteins were able to form a stable complex. A 1:1 mixture of the two proteins resulted in a single elution peak corresponding to the heterocomplex (Fig. 4*C*).

We determined the molecular mass of the HilD-HilE complex to be 52.7 ± 0.9 kDa using SEC-MALS, confirming the formation of a heterodimer (Table 2 and Fig. 4*D*). This is consistent with the observation that HilE disrupts HilD dimerization, as both shown by our MST assay and reported previously (26). We performed additional SEC-MALS runs using truncated constructs of HilD, that is, the HilD NTD construct, or HilD lacking the first 30 residues that comprise the disordered N-terminus (HilD_31-309_). The formation of a stable heterodimer was again observed, showing that HilE interacts with the HilD NTD and that neither the HilD DBD nor the N-terminus are required for binding (Figs. 4*E* and S8*B*). We also performed SEC-MALS experiments for the other *hilA* activator HilC, to investigate any potential interaction between HilC and HilE. No complex formation between HilC and HilE was observed, with two clear peaks in the UV trace corresponding to the elution of the individual proteins (Fig. S8*A* and Table S2). These results show that HilE interacts specifically with the NTD of HilD, in line with a previous study showing that HilE inhibits the DNA-binding activity of HilD, but not that of HilC (27).

**Table 2:** Molecular weight values determined from SEC-MALS

Protein sample	Oligomerization State	Molecular mass (kDa)	
		Theoretical	SEC-MALS
HilD	Dimer	70.4	70.4 ± 2.0
HilE	Monomer	16.9	19.4 ± 1.1
HilD + HilE	1:1 complex	52.1	52.7 ± 0.9
HilD_7-206_ (NTD)	Dimer	47.8	46.9 ± 1.4
NTD + HilE	Peak 1 (NTD + HilE)	40.8	40.6 ± 1.1
	Peak 2 (HilE)	16.9	24.1 ± 4.5

Calculated molecular weight values from SEC-MALS runs (Fig. 4, *D* and *E*), and the theoretical masses of the corresponding species. Standard deviation is calculated from three replicate experiments.

### HilE may directly replace one of the monomers of the HilD dimer

We modeled the HilD-HilE heterodimer complex using AlphaFold multimer (30), with the predicted structure showing that HilE directly disrupts HilD dimerization by displacing one of the two HilD molecules constituting the dimer pair (Fig. 4*F*). In all predicted models, the dimerization helix of HilD binds to the opposite face of the HilE β-barrel to that of the HilE α-helix, similar to the interactions between neighboring subunits in the hexameric structures formed by other Hcp proteins. In both the HilD homodimer and HilD-HilE heterodimer, the interface is dominated by hydrophobic interactions; however, the predicted HilD-HilE binding interface also contains several hydrogen bond and salt bridge interactions. We determined the residues forming the HilD homodimer interface using PISA (39), and calculated the buried surface area to be >935 Ǻ^2^ in all predicted models. For the HilD-HilE complex prediction, the calculated buried surface area lies between 518 and 779 Ǻ^2^ for the ten highest ranked models, suggesting that the binding interface of the HilD-HilE heterodimer may be smaller than that in the HilD homodimer.

**Figure 5:**
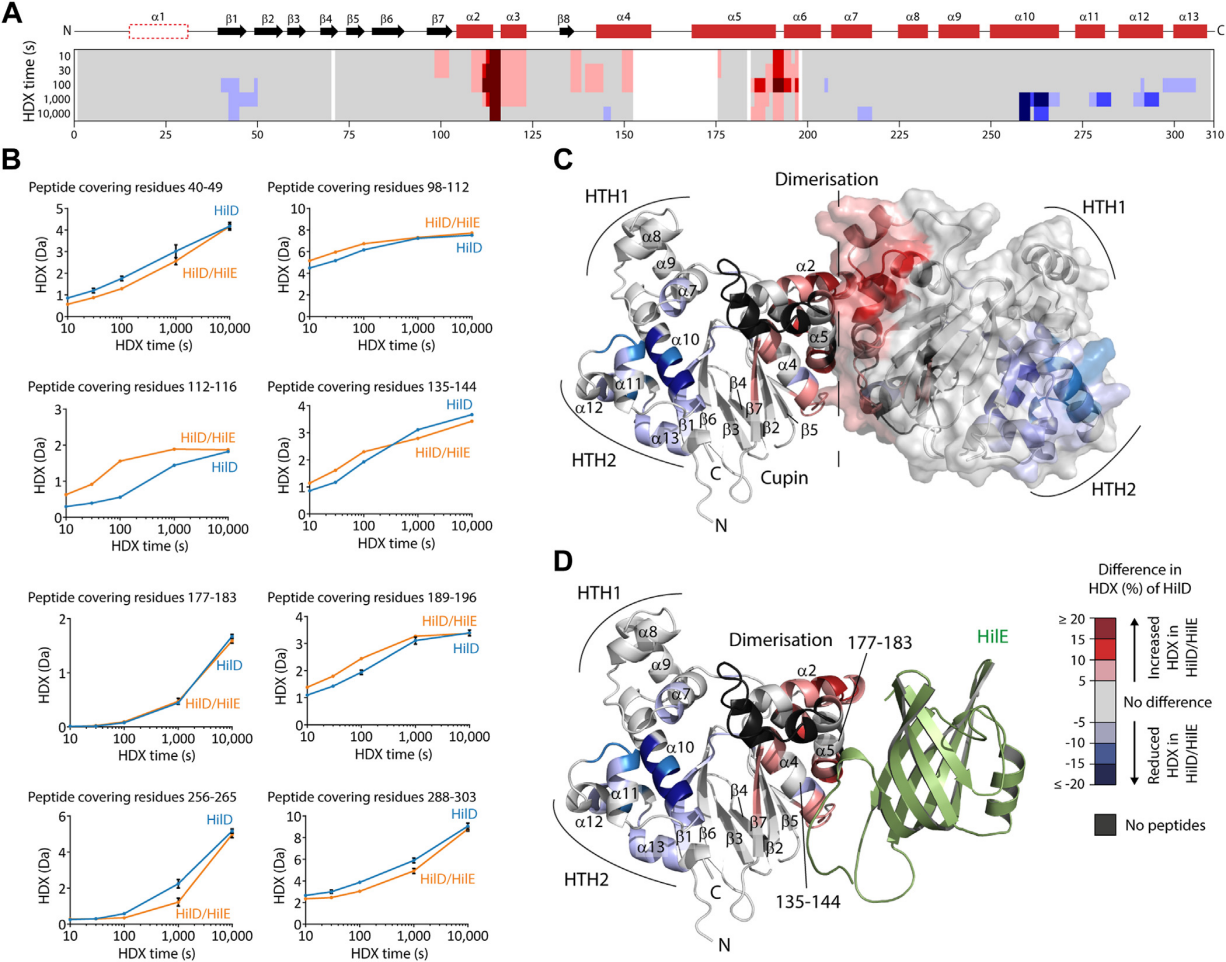
**Conformational changes of HilD in the HilD-HilE complex.***A*, the difference in HDX between the HilD-HilE complex and individual HilD projected onto the HilD amino acid sequence. Different tones of *red* and *blue* reflect increased and decreased HDX of HilD the complex. The HilD secondary structure is schematically depicted above. *B*, representative HilD peptides displaying changes in HDX. Data represent the mean ± SD of three replicates. *C* and *D*, the altered HDX of HilD in the HilD-HilE complex was projected onto a model of (*C*) the HilD-HilD homodimer or (*D*) the HilD-HilE heterodimer.

**Figure 6:**
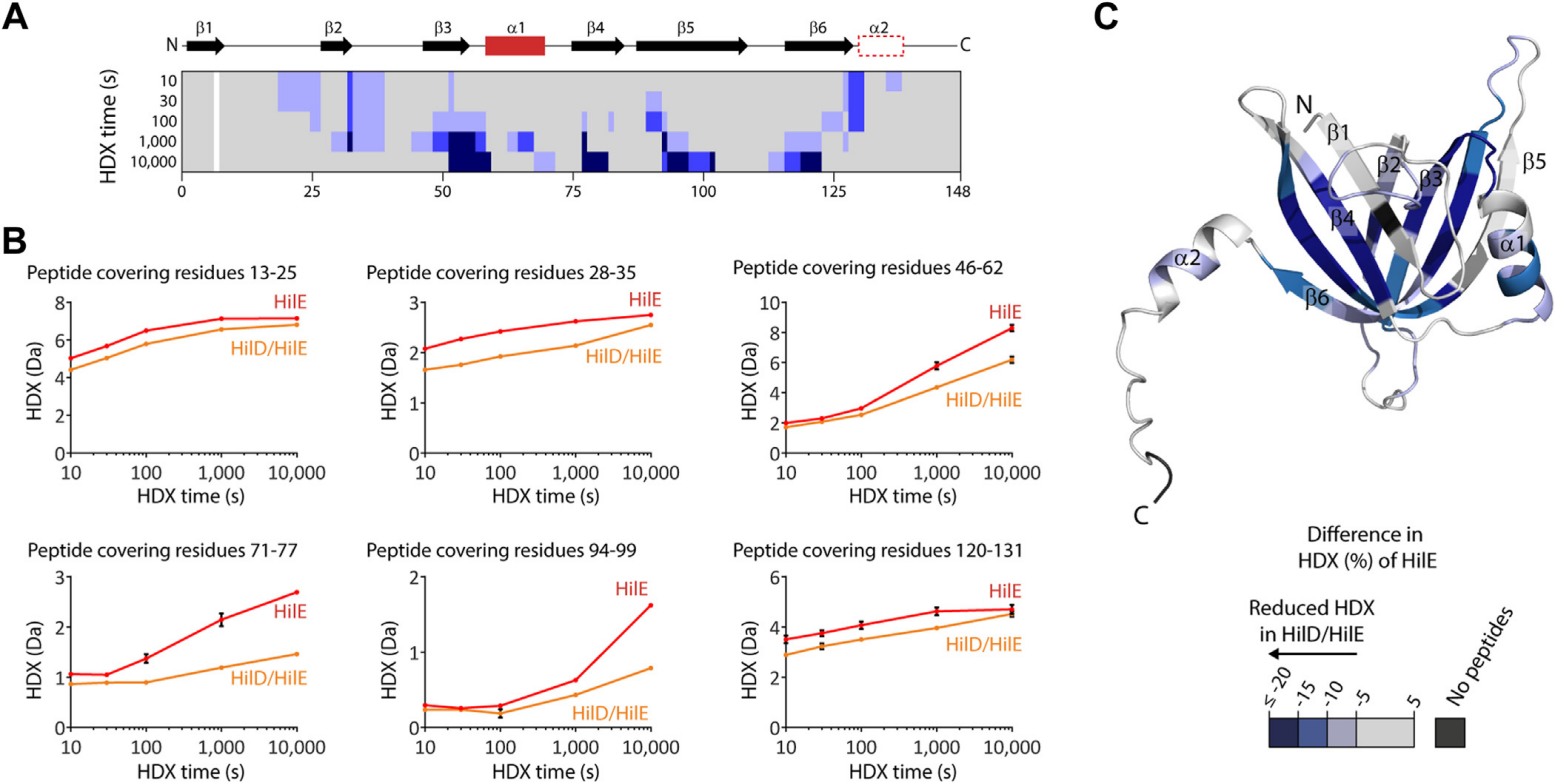
**Conformational changes of HilE in the HilD-HilE complex.***A*, the difference in HDX between the HilD-HilE complex and individual HilE projected onto the HilE amino acid sequence. Different tones of *blue* reflect decreased HDX of HilE when in complex with HilD. The HilE secondary structure is schematically depicted above. *B*, representative HilE peptides displaying changes in HDX. Data represent the mean ± SD of three replicates. *C*, the difference in HDX from (*A*) projected onto a model of HilE.

To experimentally probe our model of HilD-HilD homodimer disruption by formation of the HilD-HilE heterodimer, we performed HDX-MS experiments for both individual HilD and HilE proteins and the HilD-HilE complex (Figs. 5 and 6). Increased HDX covering most of the dimerization interface of HilD in the context of the HilD-HilE heterodimer is consistent with the notion that HilE disrupts HilD homodimerization (Fig. 5, *A*–*C*). No change though was apparent for residues 177 to 183 at the center of the HilD dimerization helix α5 (Fig. 5) and a number of peptides covering helix α4 displayed perturbed HDX, characterized by an HDX increase after short HDX incubation times but decrease at longer HDX times (representative peptide covering residues 135–144, Fig. 5*B*). Notably, this mixed HDX behavior (both increased and decreased HDX) is observed only for residues at the center of helices α4 and α5, which reflect the primary contact sites for HilE in our model of the HilD-HilE complex (Fig. 5*D*). Residues 185 to 196 (α5-α6), which are at the periphery of the predicted HilD homodimer interface, display strong increased HDX upon HilE binding, as do residues 110 to 123 (α2-α3), which constitute part of the predicted binding interface of the HilD homodimer but not that of the HilD-HilE heterodimer. This supports the prediction of a smaller interface in the HilD-HilE heterodimer, and the mixed HDX behavior at helices α4 and α5 seems to reflect the different binding modes and differences in buried surface areas of the homodimeric and heterodimeric complexes.

Decreased HDX of HilD upon HilE binding is also observed across residues 214 to 218 (α7), and at residues 41 to 50 (β1), 258 to 268 (the C-terminal end of α10) and helices α11 to α13, which comprise HTH2 and the surrounding regions. However, mutation of residues in these regions that are conserved in HilC and RtsA, but not HilD, had no effect on the binding affinity to HilE (Fig. S9, *A* and *B*). Similarly, mutation of residues in the binding pocket that were shown to be involved in the binding of LCFAs had no effect on HilE binding (Fig. S9*C*). Combined with our SEC-MALS experiments, which indicated that the DBD is not required for the HilD-HilE interaction (Fig. 4*E*), we hypothesize that these areas of decreased HDX in the DBD do not reflect the HilE binding site itself (or a part of it) on HilD, but are more likely conformational changes associated with HilE binding. Notably, these areas with reduced HDX in HilD-HilE are reminiscent of the changes induced by oleic acid (Fig. 3, *D*–*F*), which like HilE impairs HilD DNA binding ability.

We also analyzed the changes in HDX of HilE upon binding to HilD. Hereby, decreased HDX was apparent across the entire HilE domain and portions of the flanking helices (Fig. 6), implying that upon binding to HilD, conformational changes are transmitted over the β-strands and affect the entire HilE domain. Hence, this HDX experiment on HilE does not allow us to draw further conclusions on the orientation or binding interface of HilE in the HilD-HilE heterodimer.

### Two independent mechanisms of regulating HilD activity

HilE was previously shown to be dispensable for repression of HilD by *cis*-2-unsaturated LCFAs (33), as these compounds bind directly to HilD. We investigated whether competition exists between HilE and LCFAs for HilD or if a possible additive effect exists between these negative regulators to repress HilD activity.

**Figure 7:**
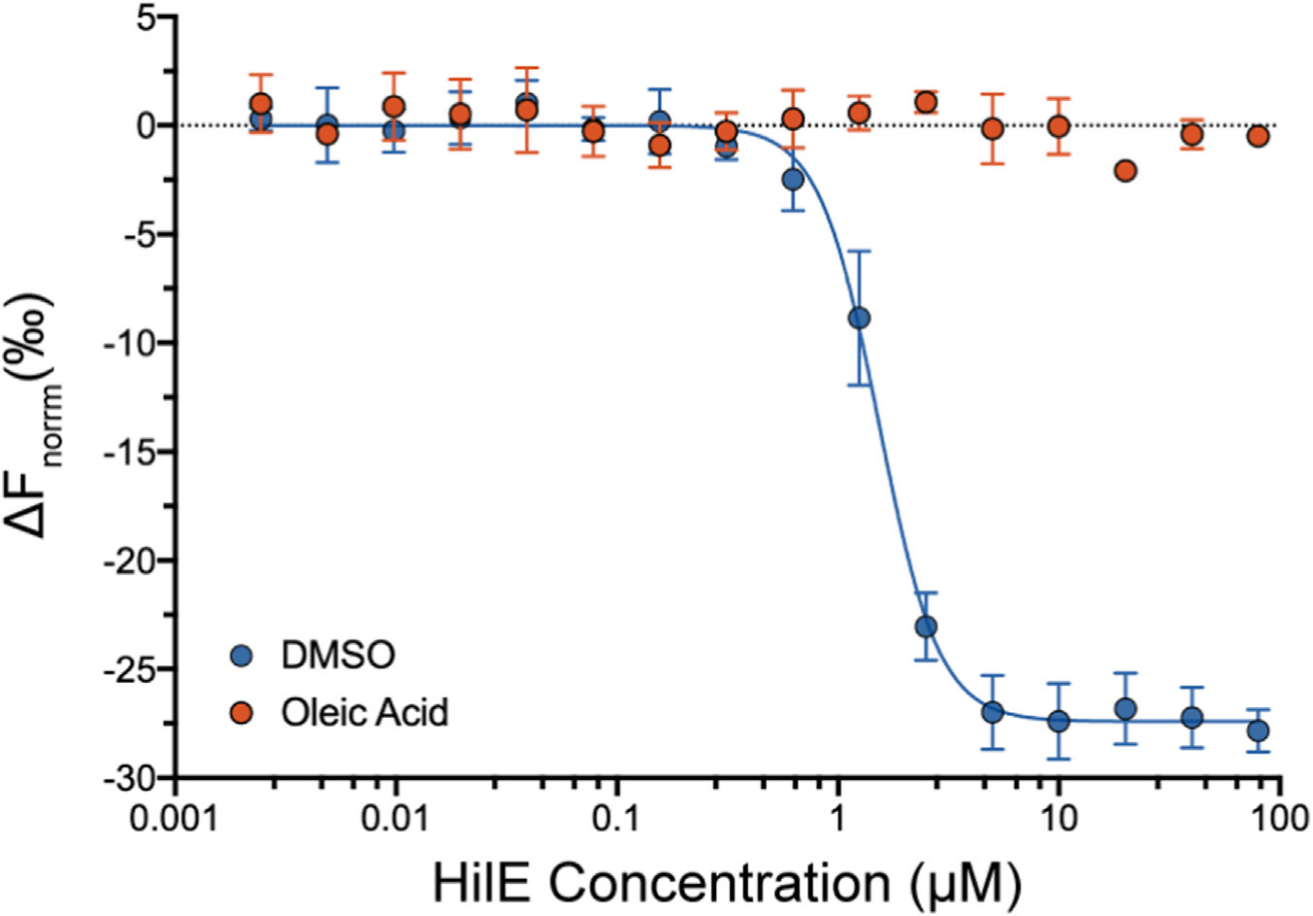
**HilE and LCFAs bind independently to HilD.** MST dose-response curves for binding of HilE to HilD. EYFP-HilD was first incubated with either 100 μM oleic acid (*orange*) or 1% DMSO (*blue*) and then increasing concentrations of HilE. Data show changes in thermophoresis at an MST on-time of 1.5 s and represents the mean ± SD of four replicates.

We first investigated the binding of HilD and HilE in the presence of oleic acid. Whilst the addition of DMSO had no significant effect on this interaction (K_d_ = 1.63 ± 0.46 μM), oleic acid prevented binding to HilE (Fig. 7). Myristoleic acid and methyl oleate, which do not bind to HilD, had no effect on HilE binding to HilD (Fig. S10, *A* and *B*). In a reverse assay setup, probing oleic acid binding to the HilD-HilE complex, changes in thermophoresis were only detected at oleic acid concentrations >40 μM, much higher than the HilE concentration (10 μM), indicating that oleic acid is unable to bind to the HilD-HilE complex (Fig. S10*C*). Taken together, this shows that only one of these regulators can bind to, and regulate, HilD at a time and that the two regulatory mechanisms exist independently of one another.

## Discussion

LCFAs regulate the function of several AraC/XylS transcription factors (40, 41), and structures of ToxT and Rns show these ligands bind to a common pocket at the interface of the two protein domains (14, 15). Using HDX-MS, we showed that the fatty acid binding mode is also conserved in HilD. Our computational model and mutational experiments indicated the interaction of fatty acids with residues E102 and R267 of HilD and, as seen for ToxT and Rns, shows that while the pocket is conserved between regulators, the specific binding residues vary. Specific interactions formed between the bound fatty acid and residues situated on both HilD domains may constrain HilD to a more stable, closed conformation and we found that binding of oleic acid increases the melting temperature of HilD. This supports the hypothesis that regulation of AraC/XylS transcription factors by fatty acids occurs *via* a common dynamic allosteric mechanism (35), inhibiting protein dimerization and subsequent binding to target DNA. HilD can bind a range of fatty acids with a chain length of at least 16 carbon atoms. Fatty acid mimetics that meet the structural requirements for binding may present an opportunity for the optimization of increasingly potent inhibitors of *Salmonella* virulence.

The activity of AraC/XylS transcription regulators may also be modulated through protein-protein interactions, and a conserved family of AraC negative regulators is widespread among pathogenetic bacteria species in which virulence genes are regulated by AraC/XylS proteins (42). The *Salmonella* negative regulator HilE is instead homologous to Hcp proteins. Our results show that unlike other characterized Hcp proteins, HilE exists predominately as a monomer, and the deletion of an extended sequence shown to be critical for the oligomerization of other Hcp family proteins supports the hypothesis that HilE diverged from an ancestral structural Hcp protein required for virulence to a regulator of such virulence genes.

HilE forms a 1:1 complex with HilD, to inhibit HilD homodimerization and prevent binding to DNA. Our results indicate that HilE interacts with the dimerization helix of HilD, directly replacing one of the HilD monomers constituting the dimer pair. LCFA-binding to HilD is expected to result in conformational changes in the dimerization helix, as reported for ToxT, restricting this helix to an orientation that is incompatible with the binding of HilE. This contrasts to *Pseudomonas aeruginosa* ExsD, which inhibits the dimerization of the AraC/XylS regulator ExsA but does not bind directly to the ExsA dimer interface (43). Our results, therefore, highlight a previously unreported mechanism for the regulation of AraC/XylS proteins, which presents an attractive prospect for the development of highly specific HilD binders. In addition to small molecules targeting the HilD-HilE complex, peptide-based inhibitors could be designed that mimic HilE binding to inhibit HilD dimerization and activity.

Binding affinity measurements showed that the HilD-HilE interaction is of higher affinity than that calculated for the homodimerization of HilD, supporting the hypothesis that HilD is bound to HilE under normal, non-invasive conditions. This repressive effect would only be overcome once HilD is expressed above the level of available HilE, by the action of positive regulators under conditions suitable for invasion (*i.e.* at the intestinal epithelium). Our results show that the mechanism of LCFA-repression of HilD is independent and mutually exclusive from that of HilE. HilD can only activate *hilA* expression when all conditions surpassing these repressive effects are met simultaneously, underlining the level of control over the expression of virulence genes to ensure efficiency of *Salmonella* pathogenesis.

## Experimental procedures

### Cloning of protein constructs for expression

Genes encoding the desired proteins were inserted into either pET-21a(+) (*hilC*, *hilE*) or pET-24a(+) (*hilD*). The *hilD* construct contained an N-terminal His_6_-SUMO tag for expression, while *hilC* and *hilE* contained an N-terminal His_6_ tag followed by a tobacco etch virus (TEV) protease cleavage site. The *hilC* and *hilE* fusion genes were synthesized and purchased from Synbio Technologies, and the pET-SUMO-HilD plasmid was a gift from Marc Erhardt.

To clone the EYFP-HilD fusion protein, the *eyfp* gene was amplified and BamHI (GGATCC) and EheI (GGCGCC) restriction sites inserted for ligation into the SUMO-HilD expression vector, into which the corresponding restriction sites were also introduced.

The His_6_-tagged HilD NTD construct was cloned using Round-the-Horn PCR, using the primer pairs HilD_NTD_fwd/rev and His-NTD_fwd/rev (Table S3) to remove the DBD and SUMO fusion tag, respectively. This construct comprised HilD residues 7 to 206, with a His_6_ tag in place of the first six N-terminal residues of the HilD sequence.

### Recombinant protein expression

Proteins were expressed in *Escherichia coli* C41(DE3) (44) (or LEMO(DE3) (45) in the case of HilE) cells using lysogeny broth (LB) medium. An overnight culture was inoculated into LB medium and grown at 37 °C until an *A*_600nm_ of 0.6 to 0.8 was reached and induced by the addition of 0.5 mM IPTG. Cells were incubated with shaking overnight at 25 °C and collected by centrifugation (11,800*g*, 4 °C).

### Protein purification

Pelleted cells were resuspended in a lysis buffer (50 mM NaH_2_PO_4_ pH 7.0, 300 mM NaCl, and 10 mM imidazole), supplemented with DNase and one cOmplete EDTA-free protease inhibitor cocktail tablet (Roche #11 873,580 001). Cells were lysed using a French press (2x, 16,000 psi) and centrifuged (95,000*g*, 1 h, 4 °C). The resulting supernatant was filtered (0.40 μm) and loaded to a Ni-NTA column. The column was washed with 20% elution buffer (50 mM NaH_2_PO_4_ pH 7.0, 300 mM NaCl, and 250 mM imidazole) and the proteins eluted with 100% elution buffer.

For HilD and EYFP-HilD, the eluted proteins were supplemented with SUMO protease (250 ng) to cleave the His_6_-SUMO tag and dialyzed overnight at room temperature (22–25 °C) against lysis buffer. The dialyzed protein was reapplied to the Ni-NTA column, equilibrated with lysis buffer. The column was washed with 25% elution buffer to elute the cleaved protein. Proteins were then further purified by SEC, using a HiLoad 26/60 Superdex 75 pg or HiLoad 26/60 Superdex 200 pg column for HilD and EYFP-HilD, respectively, equilibrated with SEC buffer (50 mM NaH_2_PO_4_ pH 7.0, 200 mM NaCl). Purified proteins were concentrated and stored in aliquots at −80 °C.

In the case of HilC and HilE, dialysis of proteins following the initial Ni-NTA column was performed overnight at 6 °C, and the protein mixture supplemented with TEV protease (1 mg) prior to dialysis. The dialyzed protein was reapplied to the Ni-NTA column as described for HilD, and column wash fractions containing the desired protein were combined and dialyzed twice against SEC buffer at 6 °C prior to storage. For the HilD NTD construct, following the initial Ni-NTA affinity purification step, the protein was concentrated and loaded to HiLoad 26/60 Superdex 75 pg column.

For HilC, a higher NaCl concentration of 500 mM was used in all purification buffers prior to dialysis into SEC buffer. For EYFP-HilD, HilD NTD and HilE, 20 mM Tris pH 8.0 was used in place of 50 mM NaH_2_PO_4_ in all buffers, and storage buffer (20 mM Tris, pH 8.0, 150 mM NaCl) used for the final SEC/dialysis purification step.

### SEC-MALS

SEC-MALS experiments were performed using a Superdex 75 Increase 10/300 Gl column (Cytiva) coupled to a miniDAWN Tristar Laser photometer (Wyatt) and a RI-2031 differential refractometer (JASCO). Fifty microliters of protein samples was loaded to the SEC column, equilibrated with SEC buffer (50 mM NaH_2_PO_4_ pH 7.0, 200 mM NaCl), and separated using a flow rate of 0.5 ml min^−1^. Data analysis was carried out with ASTRA v7.3.0.18 software (Wyatt).

### Microscale thermophoresis

All MST measurements were performed on a NanoTemper Monolith NT.115 with a Nano BLUE/RED Detector using MO.Control v1.6. MST runs were performed at 25 °C, with an excitation power of 60% (or 80% for runs using tris-NTA labeled protein) and MST power set to medium. Data were analyzed using the MO.Affinity Analysis v2.3 software, and affinity constants were calculated using the *K*_*d*_ model.

Dilution series of fatty acids were prepared in ethanol, and subsequently diluted 1:100 into 50 nM EYFP-HilD in assay buffer (20 mM Tris, pH 8.0, 150 mM NaCl), giving a constant final ethanol concentration of 1% (v/v).

For HilD dimerization and HilE binding, a two-fold serial dilution of proteins was performed in the corresponding protein storage buffer. Proteins were then mixed 1:1 with 100 nM EYFP-HilD (diluted in the corresponding storage buffer, supplemented with 0.1% (v/v) Pluronic F-127), resulting in a final assay concentration of 0.05% (v/v) Pluronic F-127 (see Supplementary information and Fig. S11 for detailed explanation).

To determine the affinity of different HilD mutants to HilE, His_6_-SUMO-HilE was labeled using the RED-tris-NTA 2nd Generation labeling kit (NanoTemper Technologies GmbH, #MO-L018). His_6_-SUMO-HilE (200 nM) was incubated with tris-NTA dye (50 nM) in assay buffer (50 mM NaH_2_PO_4_ pH 7.0, 200 mM NaCl, 0.1% (v/v) Pluronic F-127) for 30 min at room temperature. Labeled His_6_-SUMO-HilE was mixed 1:1 with a serial dilution of HilD (in SEC buffer), yielding final assay concentrations of 100 nM and 25 nM for His_6_-SUMO-HilE and the tris-NTA dye, respectively.

All samples were incubated together for 10 min at room temperature (22–25 °C), centrifuged for 5 min and loaded to standard capillaries (NanoTemper Technologies GmbH, #MO-K022). For runs using tris-NTA labeled protein, samples were instead loaded to premium capillaries (NanoTemper Technologies GmbH, #MO-K025). To investigate competitive effects of different ligands, EYFP-HilD was preincubated with the competing ligand for ≥ 10 min at room temperature, before mixing with the second ligand (of varying concentration).

### HDX-MS

Two different HDX experiments were conducted on HilD (datasets 1 and 2 in Dataset S1, respectively) To investigate the impact of oleic acid on HilD conformation (dataset 1), HilD was supplemented with 1% (v/v) of either DMSO or oleic acid (10 mM in DMSO) yielding a final concentration of 100 μM oleic acid in the sample. For experiments probing the HilD-HilE interaction (dataset 2), samples contained either individual HilD or HilE, or the HilD-HilE complex (all proteins at 25 μM final concentration), which was established prior to HDX-MS by purification using a Superdex 75 Increase 10/300 Gl column (Cytiva) equilibrated in SEC buffer (50 mM NaH_2_PO_4_/Na_2_HPO_4_ pH 7.0, 200 mM NaCl). Final assay concentrations of HilD and/or HilE in all experiments were 25 μM. All samples were stored in a cooled tray (1 °C) until measurement.

Preparation of the HDX reactions was aided by a two-arm robotic autosampler (LEAP technologies). A total of 7.5 μl of protein sample (see above) was mixed with 67.5 μl of SEC buffer, prepared with 99.9% D_2_O, to initiate the hydrogen exchange reaction. After incubation at 25 °C for 10, 30, 100, 1000 or 10,000 s, 55 μl of the HDX reaction was withdrawn and added to 55 μl of predispensed quench buffer (400 mM KH_2_PO_4_/H_3_PO_4_, pH 2.2, 2 M guanidine-HCl) kept at 1 °C. Ninety-five microliters of the resulting mixture was injected into an ACQUITY UPLC M-Class System with HDX Technology (Waters) (46). Undeuterated protein samples were prepared similarly (incubation for approximately 10 s at 25 °C) through 10-fold dilution of protein samples with water-containing SEC buffer. The injected samples were flushed out of the loop (50 μl) with H_2_O + 0.1% (v/v) formic acid (100 μl min^−1^) and guided to a protease column (2 mm × 2 cm) containing proteases immobilized to the bead material which was kept at 12 °C. For each protein state and time point, three replicates (individual HDX reactions) were digested with porcine pepsin, while another three replicates were digested with a column filled with a 1:1 mixture of protease type XVIII from *Rhizopus* spp. and protease type XIII from *Aspergillus saitoi*. In both cases, the resulting peptides were trapped on an AQUITY UPLC BEH C18 1.7 μm 2.1 × 5 mm VanGuard Pre-column (Waters) kept at 0.5 °C. After 3 min of digestion and trapping, the trap column was placed in line with an ACQUITY UPLC BEH C18 1.7 μm 1.0 × 100 mm column (Waters), and the peptides eluted at 0.5 °C using a gradient of buffers A (H_2_O + 0.1% (v/v) formic acid) and B (acetonitrile + 0.1% (v/v) formic acid) at a flow rate of 60 μl min^−1^ as follows: 0-7 min: 95-65% A; 7-8 min: 65-15% A; 8-10 min: 15% A; 10-11 min: 5% A; 11-16 min: 95% A. The eluted proteins were guided to a G2-Si high definition mass spectrometer (HDMS) with ion mobility separation (Waters), and peptides ionized with an electrospray ionization source (250 °C capillary temperature, spray voltage 3.0 kV) and mass spectra acquired in positive ion mode over a range of 50 to 2000 m/z in enhanced high definition MS (HDMS^E^) or HDMS mode for undeuterated and deuterated samples, respectively (47, 48). [Glu1]-Fibrinopeptide B standard (Waters) was employed for lock-mass correction. During separation of the peptide mixtures on the ACQUITY UPLC BEH C18 column, the protease column was washed three times with 80 μl of wash solution (0.5 M guanidine hydrochloride in 4% (v/v) acetonitrile,) and blank injections performed between each sample to reduce peptide carryover.

Peptide identification and analysis of deuterium incorporation were carried out with ProteinLynx Global SERVER (PLGS, Waters) and DynamX 3.0 softwares (Waters; https://www.waters.com/waters/library.htm?locale=en_US&lid=134832928) as described previously (49). In summary, peptides were identified with PLGS from the undeuterated samples acquired with enhanced HDMS by employing low energy, elevated energy, and intensity thresholds of 300, 100, and 1000 counts, respectively. Identified ions were matched to peptides with a database containing the amino acid sequence of HilD, HilE, porcine pepsin, and their reversed sequences with the following search parameters: peptide tolerance = automatic; fragment tolerance = automatic; min fragment ion matches per peptide = 1; min fragment ion matches per protein = 7; min peptide matches per protein = 3; maximum hits to return = 20; maximum protein mass = 250,000; primary digest reagent = non-specific; missed cleavages = 0; false discovery rate = 100. Only peptides that were identified in all undeuterated samples and with a minimum intensity of 30,000 counts, a maximum length of 30 amino acids, a minimum number of three products with at least 0.1 product per amino acid, a maximum mass error of 25 ppm and retention time tolerance of 0.5 min were considered for further analysis. Deuterium incorporation into peptides was quantified with DynamX 3.0 software (Waters). Hereby, the datasets generated with pepsin digestion or after digestions with proteases type XIII and XVIII were pooled. All spectra were manually inspected and, if necessary, peptides omitted (*e.g.*, in case of low signal-to-noise ratio or presence of overlapping peptides).

The observable maximal deuterium uptake of a peptide (see Dataset S1) was calculated by the number of residues minus one (for the N-terminal residue) minus the number of proline residues contained in the peptide. For the calculation of HDX in per cent the absolute HDX was divided by the theoretical maximal deuterium uptake multiplied by 100. To render the residue specific HDX differences from overlapping peptides for any given residue of HilD or HilE, the shortest peptide covering this residue was employed. Where multiple peptides were of the shortest length, the peptide with the residue closest to the peptide’s C-terminus was utilized.

### Cross-linking

HilD (10 μM) was first incubated with fatty acids at the indicated concentrations in SEC buffer (50 mM NaH_2_PO_4_ pH 7.0, 200 mM NaCl) for 20 min at room temperature. A final ethanol concentration of 1% (v/v) was present in all samples. HilD was then cross-linked by incubation with 0.2 mM BS^3^ (Thermo Fisher Scientific Pierce, A39266) at room temperature for 1 hour. The reaction was quenched by the addition of 50 mM Tris pH 7.5 for 15 min. Samples were analyzed using SDS-PAGE and visualized by silver staining.

### Electrophoretic mobility shift assays

EMSAs were performed using a 62 base pair dsDNA fragment of the *hilA* promoter encompassing the A1 binding site (50). dsDNA fragments were generated by boiling complementary primers together in TE buffer (10 mM Tris pH 8.0, 1 mM EDTA) at 95 °C for 10 min, before slowly cooling to room temperature. The forward primer was modified with a 5′-Cy5 fluorescent dye for detection. A total of 50 nM of labeled DNA was incubated with increasing concentrations of protein in EMSA buffer (20 mM Tris, pH 8.0, 100 mM KCl, 100 μM EDTA, and 3% (v/v) glycerol). To investigate the effect of fatty acids on HilD DNA binding, 600 nM HilD was incubated with fatty acids, at the indicated concentration, and 50 nM of labeled DNA in EMSA buffer. Fatty acids were first diluted in ethanol and diluted 1:100 into the protein sample to give a final ethanol concentration of 1% (v/v). All samples were incubated at 37 °C for 15 min, supplemented with diluted DNA loading dye, and separated on a 1.5 mm thick, 6% TBE gel at 6 °C at a constant voltage of 100 V. Gels were imaged using a ChemiDocMP imaging system (Bio-Rad Inc). Primer sequences were as follows: Fwd: ([Cyanine5]GGGAGTAAAGAAAAGACGATATCATTATTTTGCAAAAAAATATAAAAATAAGCGCACCATTA), Rev: (TAATGGTGCGCTTATTTTTATATTTTTTTGCAAAATAATGATATCGTCTTTTCTTTACTCCC).

### Nano differential scanning fluorimetry

Protein samples were heated from 20 to 90 °C, with a temperature gradient of 0.4 °C min^−1^. Melting temperatures were calculated from changes in the fluorescence ratio (350/330 nm), using PR.Stability Analysis v1.0.3 software (NanoTemper Technologies GmbH). To assess ligand-induced effects on HilD stability, fatty acids were diluted 1:100 (final assay concentration 50 μM) into 20 μM HilD (in SEC buffer) to give a final ethanol concentration of 1% (v/v). Samples were incubated for 20 min at room temperature prior to loading of standard capillaries (NanoTemper Technologies GmbH, #PR-C002).

### Mass photometry

Mass photometry (MP) measurements were performed using a Refeyn One mass photometer (Refyn Ltd, Oxford). EYFP-HilD was first diluted to 100 nM using MP buffer (50 mM NaH_2_PO_4_ pH 7.0, 100 mM NaCl), and then 1:1 with MP buffer immediately prior to measurements. Molecular mass was determined in the Refeyn DiscoverMP software provided by the manufacturer, using a standard curve of bovine serum albumin (Sigma-Aldrich) and Gel Filtration Standards (Bio-Rad), measured under identical buffer conditions.

### Protein structure prediction

The structural model of HilD (UniProt ID: P0CL08) was retrieved from the AlphaFold Protein Structure Database (51). The structure of HilE was predicted using the following publicly available prediction structure prediction servers: Alphafold2 (52), RoseTTAFold (53) and tFold (https://drug.ai.tencent.com). Complex structures of the HilD homodimer and HilD-HilE heterodimer were predicted using AlphaFold-Multimer (v2.2.0) (31). All structure models can be found in the supplementary material.

### Binding site prediction and molecular docking

System preparation and docking calculations were performed using the Schrödinger Drug Discovery suite for molecular modeling (version 2022.1). Protein−ligand complex was prepared with the Protein Preparation Wizard to fix protonation states of amino acids, add hydrogens, and fix missing side-chain atoms. All ligands for docking were drawn using Maestro and prepared using LigPrep (54) to generate the 3D conformation, adjust the protonation state to physiological pH (7.4), and calculate the partial atomic charges with the OPLS4 force field. Docking studies with the prepared ligands were performed using Glide (Glide V7.7) (55, 56) with the flexible modality of induced-fit docking with extra precision (XP), followed by a side-chain minimization step using Prime. Ligands were docked within a grid around 12 Å from the centroid of the predicted binding site pocket determined using SiteMap..

## Data availability

All data described are contained within the manuscript or available as supporting information.

## Supporting information

This article contains supporting information.

## Conflict of interest

The authors declare that they have no conflicts of interest with the contents of this article.
