# Supplementary material for: HilE represses the activity of the Salmonella virulence regulator HilD via a mechanism distinct from that of intestinal long-chain fatty acids
Source: J Biol Chem. 2023 Oct 27;299(12):105387. doi: 10.1016/j.jbc.2023.105387 (PMC10696396; doi:10.1016/j.jbc.2023.105387)
Supplement: Supporting Information [file mmc2.pdf]

HilE represses the activity of the *Salmonella* virulence regulator HilD via a mechanism distinct from that of intestinal long-chain fatty acids.

Joe D. Joiner, Wieland Steinchen, Nick Mozer, Thales Kronenberger, Gert Bange, Antti Poso, Samuel Wagner, Marcus D. Hartmann.

List of Supporting Material Contained within this Document

Supplementary Text. Effect of Pluronic on MST binding affinity measurements.

Table S1. Affinity values for the binding of LCFAs to HilD in presence of Pluronic.

Table S2. Molecular weight values determined from additional SEC-MALS runs.

Table S3. Primers used for Round-the-Horn PCR.

Supplementary Figures S1-S11.

Other Supporting Material for this Manuscript

Dataset S1. Overview HDX-MS conditions and deuterium incorporation of peptides.

Dataset S2. Predicted AlphaFold Multimer structures of the HilD-HilD homodimer.

Dataset S3. Predicted AlphaFold Multimer structures of the HilD-HilE heterodimer.

Effect of Pluronic on Calculated Binding Affinities from MST Measurements

Due to encountered issues with protein aggregation and adsorption to capillaries in MST assays, Pluronic F-127 was initially added to MST buffers, to a final assay concentration of 0.05%. Whilst this had only a minor effect on the calculated binding affinities for HilD homodimerisation or HilD/HilE binding affinity (Fig. S11), we found that the presence of Pluronic resulted in much higher K_d values (and underestimation of the affinity) for the binding of fatty acids (Table S1, Fig. S4). Hence, Pluronic was omitted from MST assay buffer for runs determining affinities of LCFAs to HilD. The trends in binding affinity of different LCFAs in the presence of Pluronic are similar to those obtained without Pluronic in both MST and EMSA experiments. We hypothesise that this effect is due to the incorporation of the LCFAs into micelles formed by Pluronic in aqueous buffers, reducing the amount of free LCFAs in solution to below the expected concentration, and resulting in the underestimation of binding affinities.

Table S1. Affinity values for the binding of LCFAs to HilD in presence of Pluronic.

Lipid	Shorthand Nomenclature	K_d ± SD (μM)	
Myristoleic Acid	9Z-14:1	566.7	± 59.5
Palmitoleic Acid	9Z-16:1	58.53	± 4.86
Oleic Acid	9Z-18:1	48.06	± 5.27
Gadoleic Acid	9Z-20:1	26.68	± 3.13
Erucic Acid	13Z-22:1	14.71	± 2.19
Nervonic Acid	15Z-24:1	17.99	± 1.84
Methyl Oleate	9Z-18:1	738.5	± 276.1

K_d values were calculated from changes in normalised fluorescence (ΔF_{norm}) at an MST on-time of 1.5 seconds with increasing ligand concentrations, from at least 3 replicates. Replicates were merged and the standard deviation calculated using the MO.Affinity Analysis v2.3 software (NanoTemper Technologies GmbH).

Table S2. Molecular weight values determined from additional SEC-MALS runs (shown in Fig. S8).

Protein Sample	Oligomerisation State	Molecular Mass (kDa)		
		Theoretical	SEC-MALS	
HilC	Dimer	68.0	69.3	± 0.9
HilE	Monomer	16.9	23.2	± 3.6
HilC + HilE	Peak 1 (HilC)	68.0	70.4	± 0.6
	Peak 2 (HilE)	16.9	24.2	± 1.6
HilD$_{31\text{-}309}$	Dimer	63.6	62.4	± 0.6
HilD$_{31\text{-}309}$ + HilE	1:1 complex	48.8	48.9	± 0.2
HilD S216I/S218D	Dimer	70.4	67.8	± 1.1
HilD S216I/S218D + HilE	1:1 complex	52.1	52.5	± 0.6
HilD K279M	Dimer	70.4	69.4	± 0.7
HilD K279M + HilE	1:1 complex	52.1	52.9	± 0.9

Table 3. Primers used for Round-the-Horn PCR.

Primer	Sequence
HilD_NTD_fwd	5'-TAAAAGCTTGCGGCCGCACTCGAG-3'
HilD_NTD_rev	5'-CGTTATCTGAGCCGAGCTAAGGATGATC-3'
His-NTD_fwd	5'-GTAAGTAATAGTCATCAGCGTCCTGC-3'
His-NTD_rev	5'-CGAACCATGGTGATGATGGTGATGA-3'

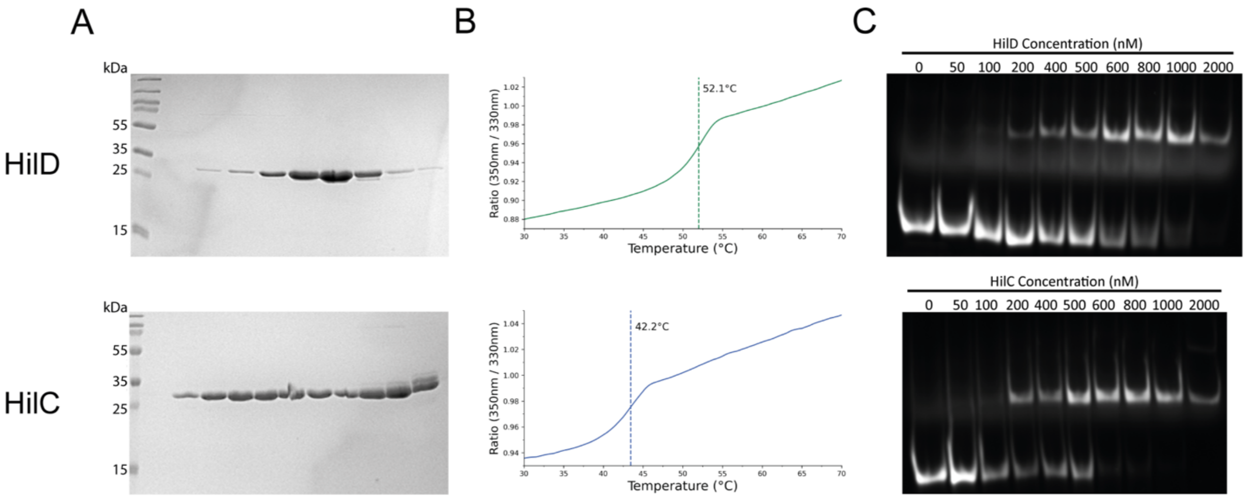

Figure S1. Biophysical characterisation of HilD and HilC. (**A**) Coomassie-stained SDS-PAGE gel for fractions eluted from the final purification step of HilD (top) and HilC (bottom). (**B**) NanoDSF unfolding profile of HilD (green, top) and HilC (blue, bottom). A concentration of 20 µM was used for both proteins. (**C**) EMSAs showing binding of HilD (top) and HilC (bottom) to the *hilA* promoter. All lanes contain 50 nM of a DNA fragment encompassing the A1 HilD binding site within the promoter and increasing protein concentrations, as indicated. DNA is labelled with a Cy5 fluorophore at the 5’ end of the forward strand for image detection.

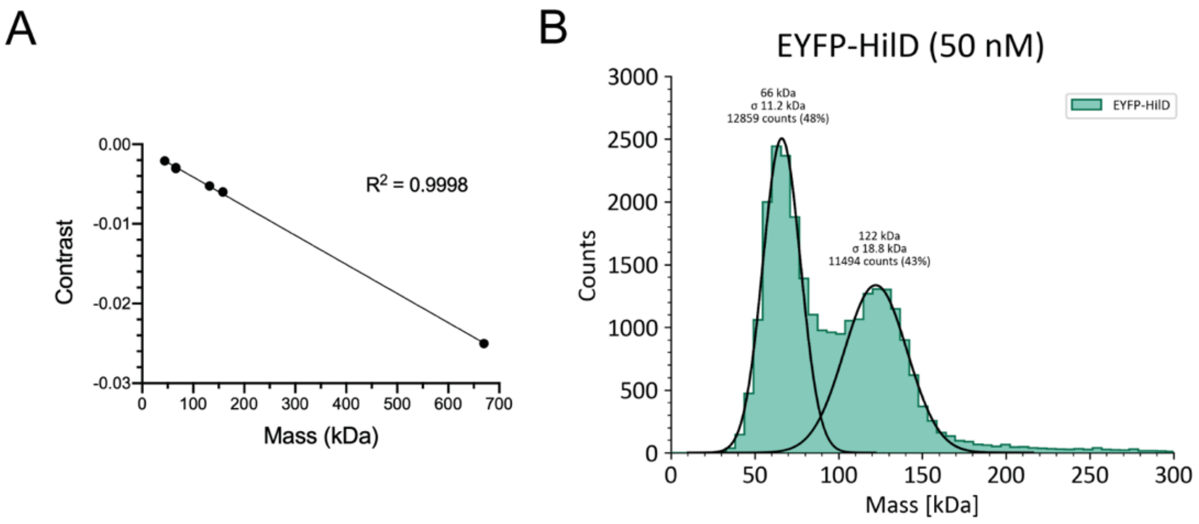

Figure S2. EYFP-HilD exists as a mixture of monomers and dimers at concentrations used for MST assays. (**A**) Mass calibration curve composed of BSA (66 and 132 kDa), ovalbumin (44 kDa), γ-globulin (158 kDa) and thyroglobulin (670 kDa). (**B**) Mass photometry mass distribution plot of EYFP-HilD (50 nM). Data is a cumulative distribution from 8 individual measurements. The number of landing events (counts) is displayed as a histogram, along with the peaks fitted by Gaussian curves. Calculated molecular weight values, standard deviation (σ) and the number of events within the Gaussian fit are displayed.

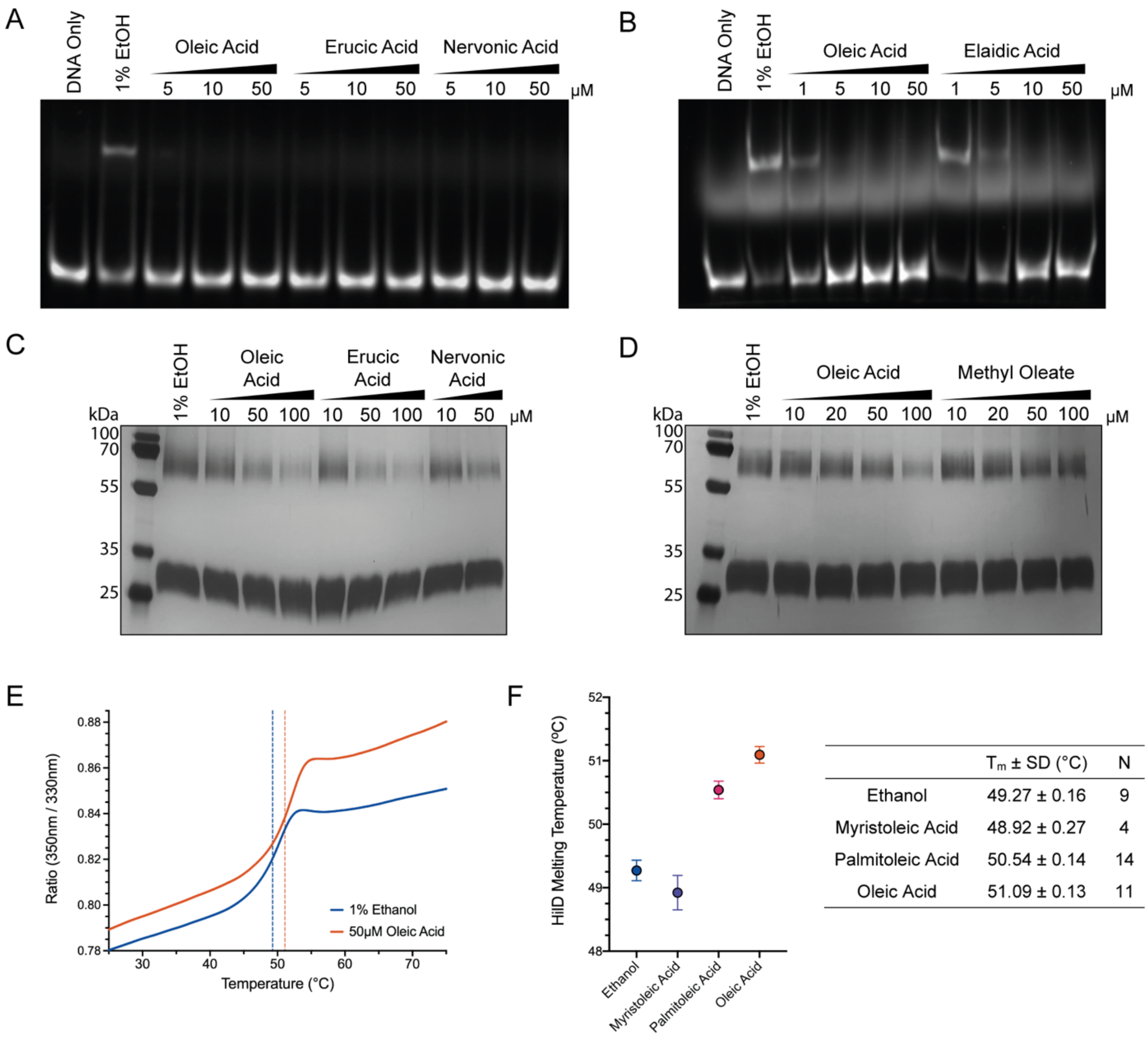

	T_m ± SD (°C)	N
Ethanol	49.27 ± 0.16	9
Myristoleic Acid	48.92 ± 0.27	4
Palmitoleic Acid	50.54 ± 0.14	14
Oleic Acid	51.09 ± 0.13	11

Figure S3. LCFAs stabilise the monomeric form of HilD. (**A-B**) EMSAs showing the inhibition of HilD DNA binding by LCFAs. HilD (600 nM) was incubated with *hilA* promoter DNA (50 nM) and LCFAs at the indicated concentrations. (**C-D**) BS^3 cross-linking of HilD in the presence of different fatty acids. HilD (10 μM) was incubated with fatty acids (or ethanol) at the indicated concentrations prior to crosslinking with BS^3. (**E**) NanoDSF unfolding profiles of HilD (20 μM) when incubated with either 1% ethanol (blue) or 50 μM oleic acid (orange). Traces show the average fluorescent readout obtained from N technical replicates, as listed in (F). Dashed vertical lines indicate the calculated HilD melting temperature for each sample. (**F**) Melting temperature of HilD in the presence of different LCFAs. Data represent mean ± SD of N technical replicates.

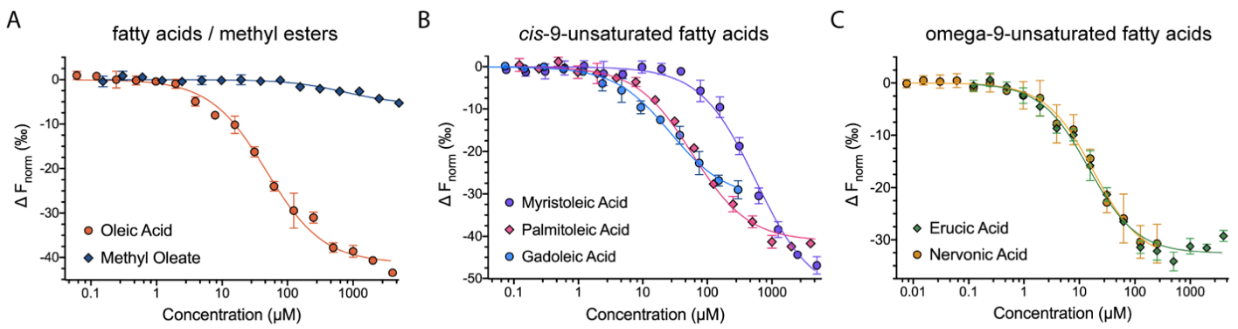

Figure S4. Dose-response curves for LCFA binding to HilD in presence of Pluronic. MST binding curves for fatty acid binding to HilD: (**A**) oleic acid and methyl oleate; (**B**) *cis*-9-unsaturated fatty acids; (**C**) omega-9- unsaturated fatty acids. Data represent K_d ± SD calculated from at least 3 replicates. Calculated affinities are displayed in Table S1.

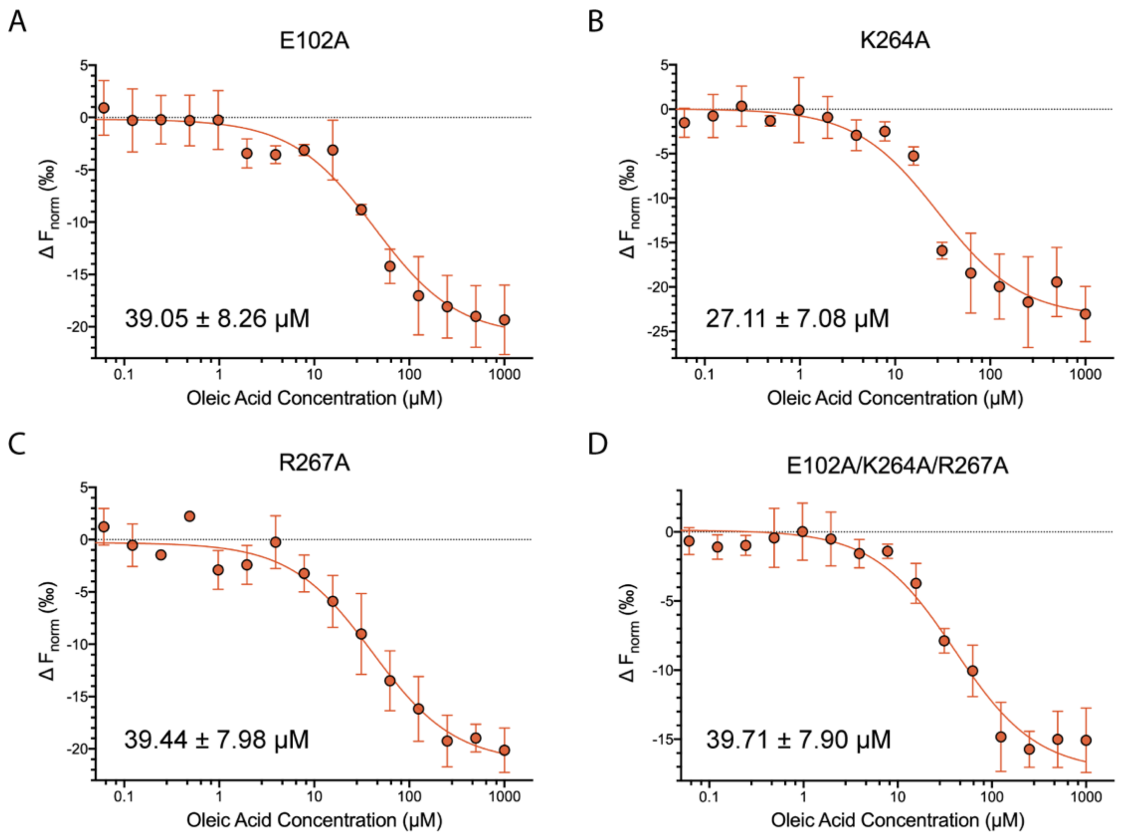

Figure S5. Dose-response curves for oleic acid binding to HilD mutants. MST binding curves for oleic acid binding to HilD mutants: (**A**) E102A, (**B**) K264A, (**C**) R267A, (**D**) E102A/K264A/R267A. Calculated K_d affinity values are shown in μM. Data represent mean ± SD from 4 replicates.

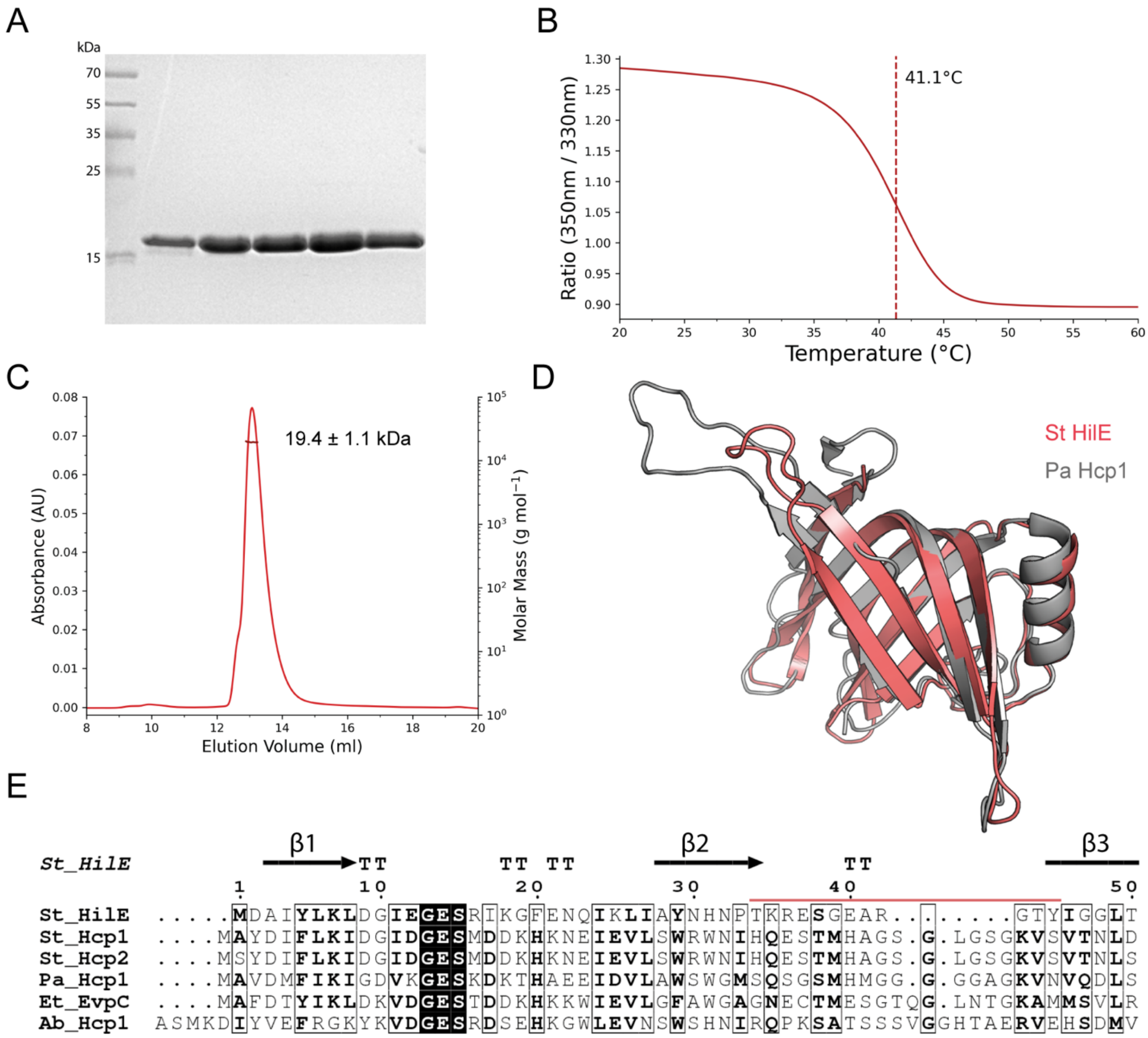

Figure S6. HilE exists exclusively as a monomer in solution. (**A**) Coomassie-stained SDS-PAGE gel for fractions eluted from the final purification step of HilE. (**B**) NanoDSF unfolding profile of HilE (1 mg ml^{-1}). (**C**) SEC-MALS elution profile of HilE. HilE (100 μM) was loaded to a S75 10/300 increase column, and absorbance was constantly monitored at 280 nm. Molecular mass of the eluted protein was calculated from the static light scattering. (**D**) Structural alignment of HilE (tfold prediction, red) with *Pseudomonas aeruginos* Hcp1 (PDB: 1Y12; grey). (**E**) Multiple sequence alignment of Hcp proteins, identified using HHPred, was performed using ClustalΩ and highlights the shortened length of the loop in HilE (residues constituting the loop in HilE are marked by the red line). β -strands, as predicted in the structure of HilE, are denoted by the arrows above the alignment.

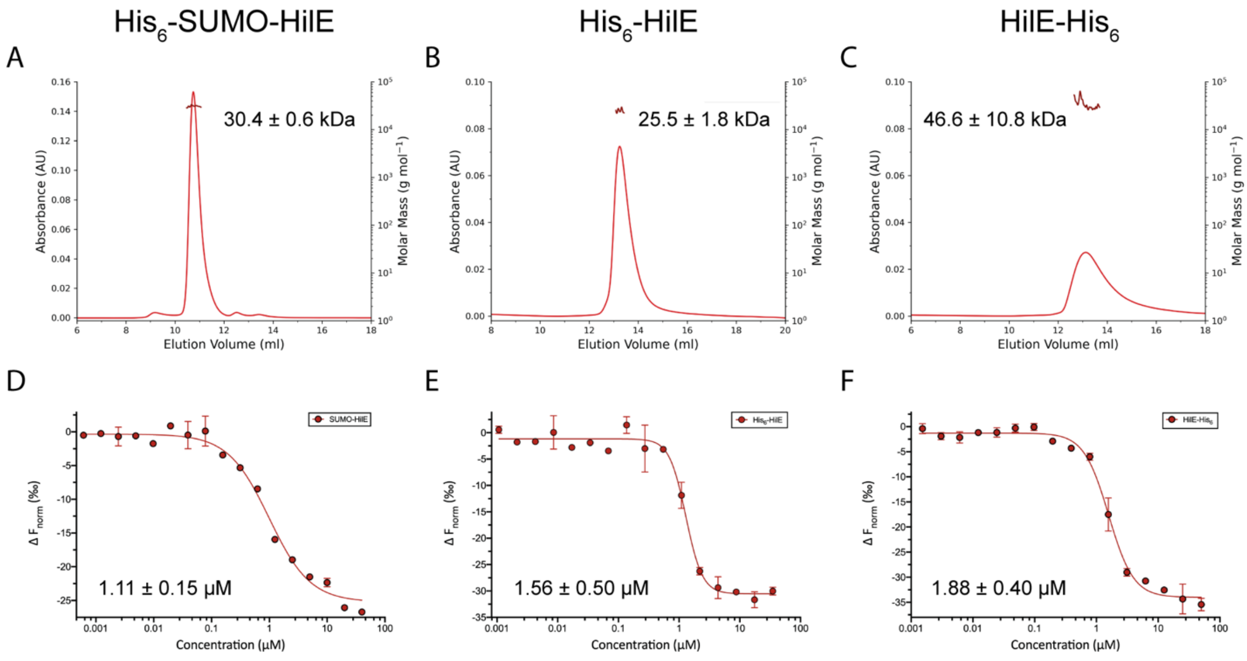

Figure S7. Oligomerisation and activity of additional purified HilE constructs. (**A-C**) SEC-MALS elution profiles of the three purified HilE constructs. Molecular weight values from light scattering measurements were calculated from three replicate runs for each construct. (**D-F**) Corresponding dose-response binding curves for the binding of each of the HilE constructs to EYFP-HilD in an MST assay. K_d affinity values were calculated from two repeat measurements and an MST on-time of 1.5 seconds used for analysis.

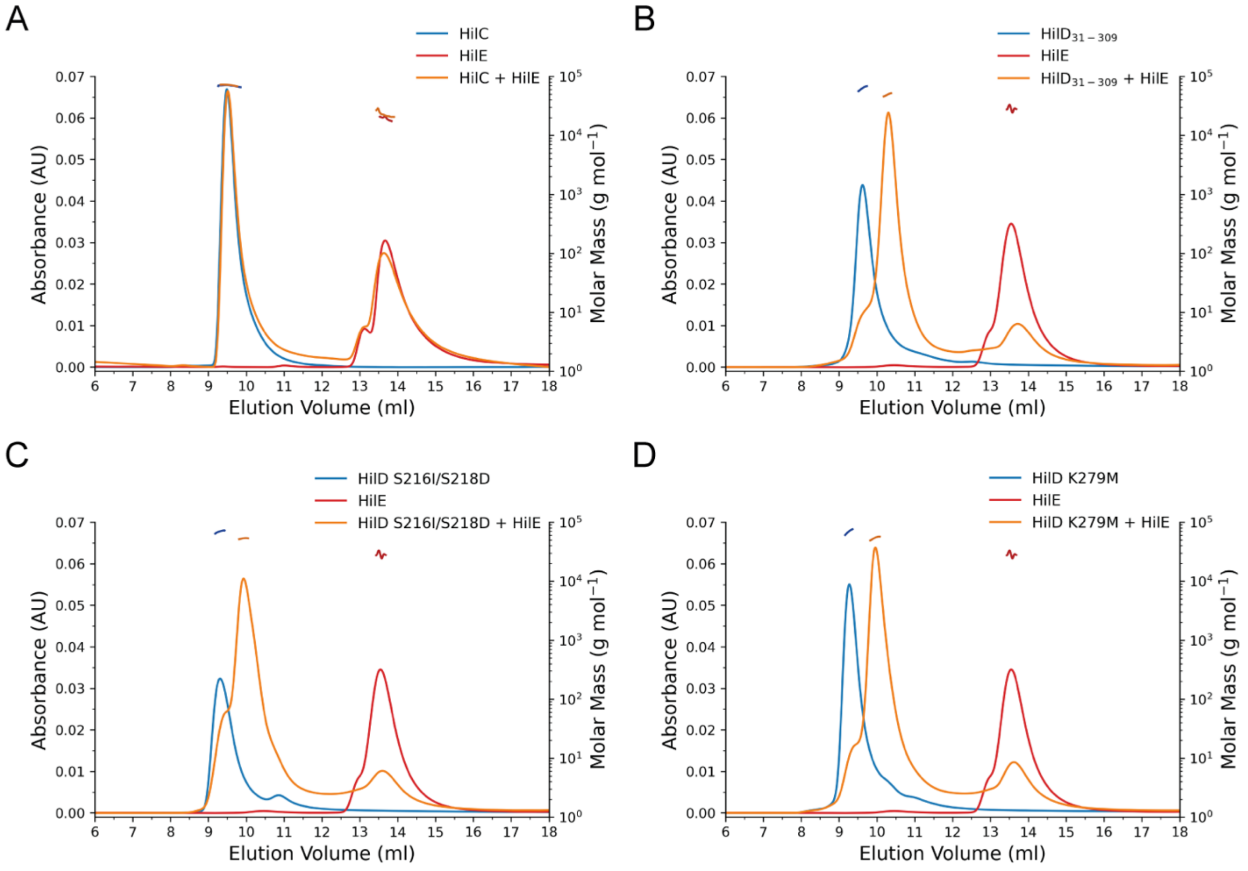

Figure S8. Additional SEC-MALS experiments. Analogous SEC-MALS experiments were performed for HilE and (**A**) HilC, (**B**) HilD construct lacking the disordered N-terminus (HilD$_{31-309}$), (**C**) HilD S216D/S218I, (**D**) HilD K279M. Protein concentrations of 50 μM were used for all samples, due to the lower solubility and observed precipitation of HilC and HilD truncations at higher concentrations. Molecular weight values were calculated from 3 repeat experiments and are displayed in Table S2.

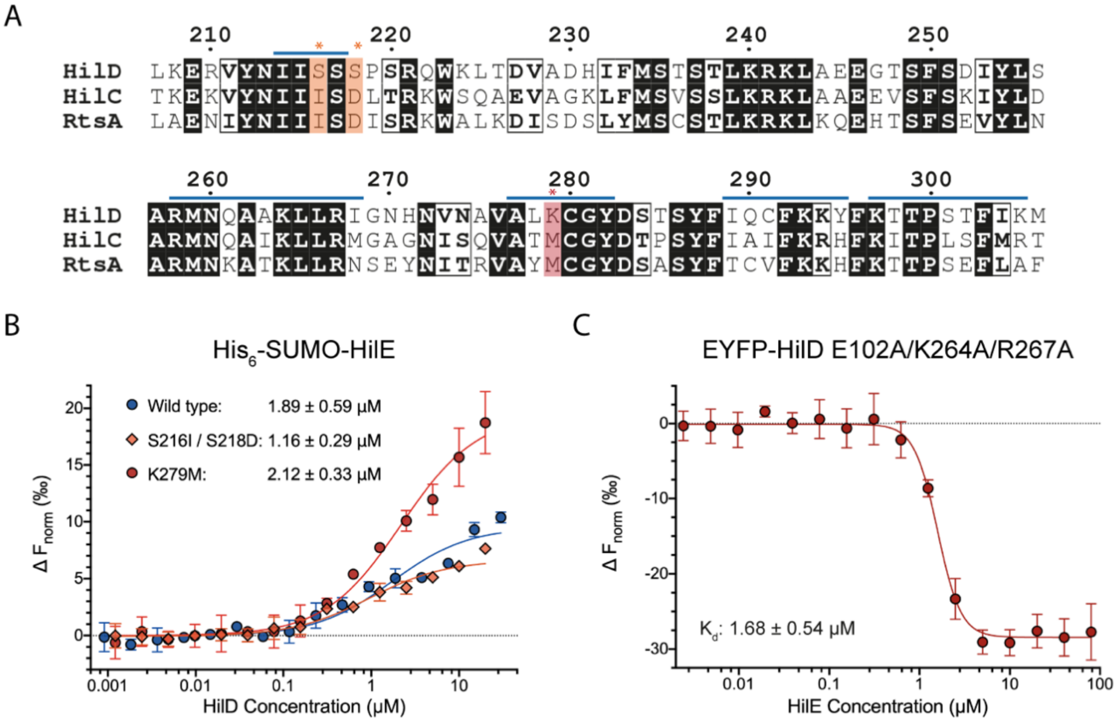

Figure S9. Mutations in the HilD fatty acid binding pocket or DNA-binding domain do not affect binding affinity to HilE. (**A**) Sequence alignment of the DNA-binding domains of HilD, HilC and RtsA, performed using ClustalΩ. Residues showing decreased HDX upon HilE binding are denoted by blue lines. HilD residues were selected for mutational analysis based on conservation in HilC and RtsA, but not HilD, and are highlighted. (**B**) MST dose-response curves for the binding of HilD mutants to HilE. His_6-SUMO-HilE (100 nM) was labelled using the RED-tris-NTA dye and incubated with increasing concentrations of each of the HilD mutants. (**C**) MST dose-response curve for the binding of HilE to the EYFP-HilD E102A/K264A/R267A triple mutant. In both (**B**) and (**C**), K_d values were calculated from changes in thermophoresis at 1.5 seconds on-time and data represent the mean ± SD of 3 replicates.

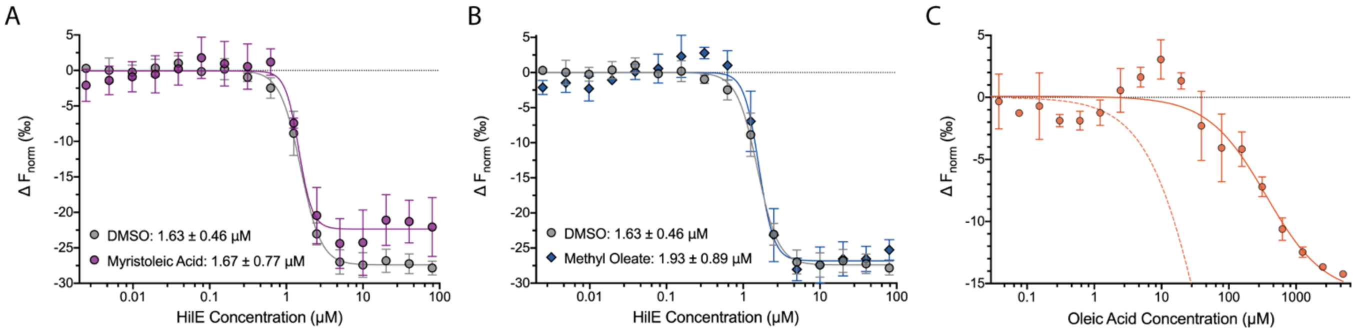

Figure S10. (Related to Fig. 7). Binding of HilD and HilE in presence of LCFAs. (**A-B**) MST dose-response curves for binding to HilE to HilD, at an MST on-time of 1.5 seconds. EYFP-HilD was incubated with 100 µM of either (**A**) myristoleic acid (purple) or (**B**) methyl oleate (blue), followed by varying concentrations of HilE. Binding of HilD and HilE after incubation with 1% DMSO (grey) is shown for reference. Data represent the mean ± SD of n=3 (methyl oleate) or n=4 (DMSO, myristoleic acid) replicates. (**C**) Oleic acid was titrated against a complex of HilD-HilE, constituted by mixing EYFP-HilD (50 nM) with HilE (10 µM). The dashed orange line shows the fitted binding curve for oleic acid binding to HilD alone (in the presence of Pluronic, see Fig. S4). Data shows changes in thermophoresis at an MST on-time of 1.5 seconds and represents the mean ± SD of 4 replicates.

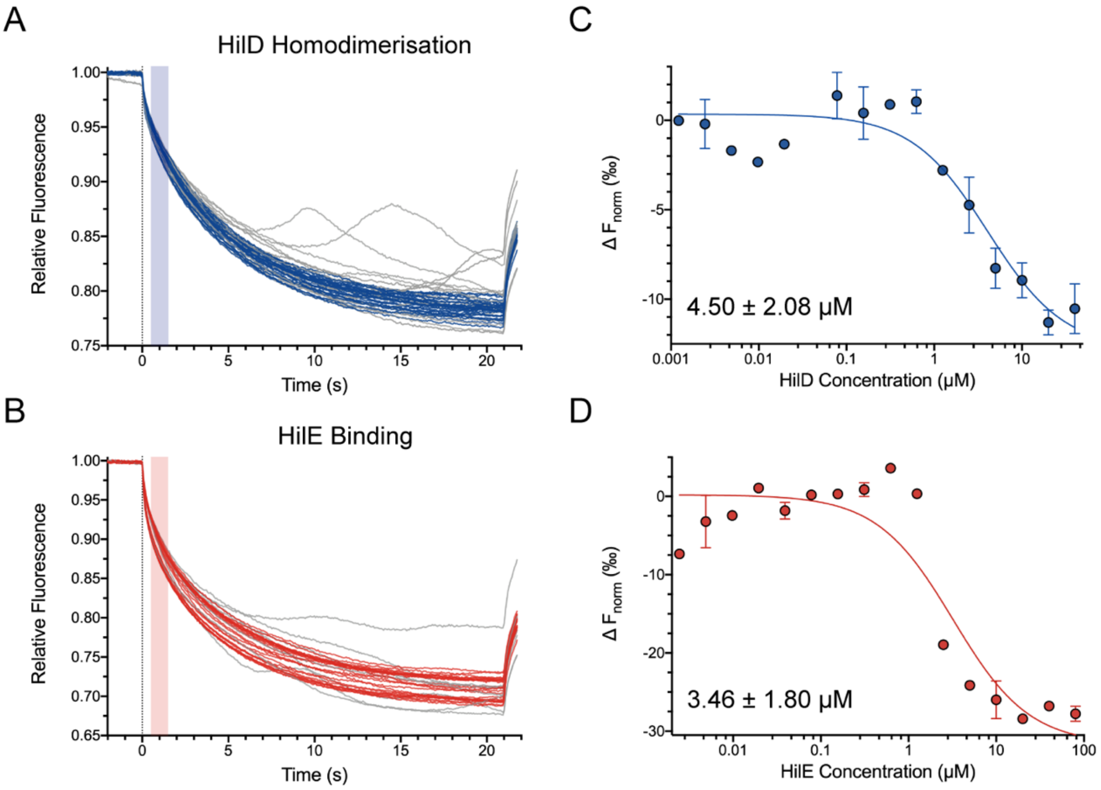

Figure S11. Pluronic does not affect HilD homodimerisation or HilE binding MST measurements. (**A-B**) Raw MST traces for (**A**) HilD homodimerisation and (**B**) HilE binding to HilD in the absence of Pluronic. Data are shown for n=3 (A) or n=2 (B) replicate MST runs. Individual MST traces for samples that showed aggregation or adsorption are coloured in grey and were excluded from subsequent data analysis. (**C-D**) Dose-response plots for (**C**) HilD homodimerisation and (**D**) HilE binding. K_d values were determined from changes in thermophoresis at an MST on-time of 1.5 seconds, highlighted by the shaded region in (A) and (B). Data represents the mean ± SD of n=3 (C) or n=2 (D) replicates, calculated from the MST traces shown in (A) and (B), respectively.
